# Extremely rare variants reveal patterns of germline mutation rate heterogeneity in humans

**DOI:** 10.1038/s41467-018-05936-5

**Published:** 2018-09-14

**Authors:** Jedidiah Carlson, Adam E. Locke, Matthew Flickinger, Matthew Zawistowski, Shawn Levy, Richard M. Myers, Michael Boehnke, Hyun Min Kang, Laura J. Scott, Jun Z. Li, Sebastian Zöllner, The BRIDGES Consortium, Devin Absher, Huda Akil, Gerome Breen, Margit Burmeister, Sarah Cohen-Woods, William G. Iacono, James A. Knowles, Lisa Legrand, Qing Lu, Matthew McGue, Melvin G. McInnis, Carlos N. Pato, Michele T. Pato, Margarita Rivera, Janet L. Sobell, John B. Vincent, Stanley J. Watson

**Affiliations:** 1 Department of Computational Medicine & Bioinformatics, University of Michigan, Ann Arbor, MI 48109 USA; 2 McDonnell Genome Institute & Department of Medicine, Washington University, St. Louis, MO 63108 USA; 3 Department of Biostatistics, University of Michigan, Ann Arbor, MI 48109 USA; 4 HudsonAlpha Institute for Biotechnology, Huntsville, AL 35806 USA; 5 Department of Human Genetics, University of Michigan, Ann Arbor, MI 48109 USA; 6 Department of Psychiatry, University of Michigan, Ann Arbor, MI 48109 USA; 7 Molecular & Behavioral Neuroscience Institute, University of Michigan, Ann Arbor, MI 48109 USA; 8 MRC Social Genetic and Developmental Psychiatry Centre, Institute of Psychiatry Psychology and Neuroscience, King’s College London, London, UK; 9 School of Psychology, Flinders University, Adelaide, South Australia Australia; 10 Department of Psychology, University of Minnesota, Minneapolis, MN 55414 USA; 11 Department of Psychiatry and the Behavioral Sciences, University of Southern California, Los Angeles, CA 90033 USA; 12 Department of Epidemiology and Biostatistics, Michigan State University, East Lansing, MI 48824 USA; 13 SUNY Downstate Medical Center, Brooklyn, NY 11203 USA; 14 Department of Psychiatry, SUNY Downstate Medical Center, Brooklyn, NY 11203 USA; 15 Molecular Neuropsychiatry and Development Laboratory, Campbell Family Mental Health Research Institute, Toronto, ON Canada

## Abstract

A detailed understanding of the genome-wide variability of single-nucleotide germline mutation rates is essential to studying human genome evolution. Here, we use ~36 million singleton variants from 3560 whole-genome sequences to infer fine-scale patterns of mutation rate heterogeneity. Mutability is jointly affected by adjacent nucleotide context and diverse genomic features of the surrounding region, including histone modifications, replication timing, and recombination rate, sometimes suggesting specific mutagenic mechanisms. Remarkably, GC content, DNase hypersensitivity, CpG islands, and H3K36 trimethylation are associated with both increased and decreased mutation rates depending on nucleotide context. We validate these estimated effects in an independent dataset of ~46,000 de novo mutations, and confirm our estimates are more accurate than previously published results based on ancestrally older variants without considering genomic features. Our results thus provide the most refined portrait to date of the factors contributing to genome-wide variability of the human germline mutation rate.

## Abstract (web summary)

Germline mutation rate is a critical parameter in the study of genetics and evolution. Here, Carlson et al. infer fine-scale patterns of human mutation rate heterogeneity by analyzing ~36 million singleton variants from 3560 whole-genome sequences.

## Introduction

Germline mutagenesis is a fundamental biological process, and a major source of all heritable genetic variation (see Segurel et al.^1^ for a review). Mutation rate estimates are widely used in genomics research to calibrate variant calling algorithms^2^, infer demographic history^3^, identify recent patterns of genome evolution^4^, and interpret clinical sequencing data to prioritize likely pathogenic mutations^5^. Although mutation is an inherently stochastic process, the distribution of mutations in the human genome is not uniform, and is correlated with genomic and epigenomic features, including local sequence context^6,7^, recombination rate^8^, and replication timing^9^. Hence, there is considerable interest in studying the regional variation and context dependency of mutation rates to understand the basic biology of mutational processes and to build accurate predictive models of this variability.

The gold standard for studying the germline mutation rate in humans is direct observation of de novo mutations from family-based whole-genome sequencing (WGS) data^9–12^. These studies have produced accurate estimates of the genome-wide average mutation rate (~1 − 1.5 × 10^−8^ mutations per base pair per generation) and uncovered some of the mutagenic effects of genomic features. However, the inherently low-germline mutation rate means family-based WGS studies detect only 40–80 de novo mutations per trio sequenced^9,10,12^, making it difficult to accumulate a dataset large enough to precisely estimate mutation rates and spectrum at a fine scale and identify factors that explain genome-wide variability in mutation rates.

Other data sources for studying mutation patterns include between-species substitutions or within-species polymorphisms^7,8,13–16^. However, because these variants arose hundreds or thousands of generations ago, their distribution patterns along the genome have been influenced by many evolutionary forces, such as natural selection and GC-biased gene conversion (gBGC), a process in which recombination-induced mismatches are preferentially repaired to G/C base pairs, resulting in an overabundance of common A/T-to-G/C variants^11,17,18^. A further complication of estimating mutation rates with ancestrally older variants is that the endogenous mutation mechanisms themselves have likely evolved over time^19^. Hence, patterns of variation observed among these data may not necessarily reflect ongoing mutation processes in the present-day population. To minimize the confounding effects of selection, studies that estimated mutation rates from these data tended to focus on intergenic noncoding regions of the genome, which are less often the target of selective pressure. Nevertheless, even putatively neutral loci may be under some degree of selection^20–22^, and are susceptible to the confounding effects of gBGC and evolving mutation processes. Consequently, these processes bias the resulting distribution of variation, making it difficult to determine which trends are attributable to the initial mutation processes, and which to subsequent evolutionary factors.

We, therefore, adopt an approach that relies exclusively on extremely rare variants (ERVs) to study innate mutation patterns across the genome. Here, we exploit a collection of ~35.6 million singleton variants discovered in 3560 sequenced individuals from the Bipolar Research in Deep Genome and Epigenome Sequencing (BRIDGES) study of bipolar disorder (corresponding to a minor allele frequency of 1/7120 = 0.0001404 in our sample). Compared to between-species substitutions or common SNVs, these ERVs are extremely young on the evolutionary timescale (in a comparably sized European sample, one study estimated the expected age of a singleton to be 1244 years^23^), making them much less likely to be affected by evolutionary processes other than random genetic drift^1,11,17,24^. ERVs thus represent a relatively unbiased sample of recent mutations and are far more numerous than de novo mutations collected in family-based WGS studies.

Our results show that mutation rate heterogeneity is primarily dependent on the sequence context of adjacent nucleotides, confirming the findings of previous studies^7,9,25^. However, we demonstrate that our ERV-derived mutation rate estimates can differ substantially from estimates based on ancestrally older variants. Evaluating these differences in an independent dataset of ~46,000 de novo mutations, collected from two published family-based WGS studies^9,12^, we find that ERV-derived estimates yield a significantly more accurate portrait of present-day germline mutation rate heterogeneity. We further refine these estimates of context-dependent mutability by systematically estimating how mutation rates of different sequence motifs are influenced by genomic features in wider surrounding regions, including replication timing, recombination rate, and histone modifications. Remarkably, we find that the direction of effect for some genomic features depends on the actual sequence motif surrounding the mutated site, underscoring the importance of jointly analyzing sequence context and genomic features. Accounting for these granular effects of the genomic landscape provides even greater accuracy in describing patterns of variation among true de novo mutations. Our results suggest that trends of variation throughout the genome are shaped by a diverse array of context-dependent mutation pathways. This high-resolution map of mutation rate estimates, along with estimates of the mutagenic effects of genomic features, is available to the community as a resource to facilitate further study of germline mutation rate heterogeneity and its implications for genetic evolution and disease.

## Results

### ERV data source and quality control

In the BRIDGES study, we sequenced the genomes of 3716 unrelated individuals of European ancestry to an average diploid-genome coverage of 9.6×. We identified and removed 156 samples which appeared to be technical outliers, resulting in a final call set of 35,574,417 autosomal ERVs from 3560 individuals (Methods). Due to the relatively low coverage of our sample, we likely failed to detect millions more ERVs—a recent study^26^ estimated the discovery rate for singletons in a sample of 4000 whole genomes at 10× coverage to be ~65–85%. Quality control measures indicate that the ERVs we detected are high quality, with a transition/transversion (Ts/Tv) ratio of 2.00, within the commonly observed range for single nucleotide variants (SNVs) from WGS data^27^ (Supplementary Table 1). Application of the 1000G strict accessibility mask^28^ (which delineates the most uniquely mappable genomic regions) or a more stringent mapping quality score filter (MQ > 56) did not appreciably change the Ts/Tv ratio (1.97–2.01) (Supplementary Table 1). We estimate fewer than 3% of the 35,574,417 ERVs are false positives (Supplementary Note), similar to the validated singleton error rates of other sequencing studies using a similar technology^28–30^. In addition, we present evidence that erroneous calls among the ERVs are unlikely to be biased by motif-specific genotyping error, mapping error, or mispolarization (Supplementary Note).

### Context-dependent variability in mutation rates

The nucleotides surrounding a mutated site are a well-known predictor of variability in mutation rates across the genome^7,11,25^. The most detailed such analysis to date, by Aggarwala and Voight^7^, considered the nucleotides up to 3 positions upstream and downstream from a variant site (i.e., a 7-mer sequence context), and estimated substitution probabilities per heptameric motif using 7,051,667 intergenic SNVs observed in 379 Europeans from phase 1 of the 1000 Genomes Project (hereafter referred to as the “1000G mutation rate estimates”). These estimates have the potential problem of being derived from variants across the entire frequency spectrum: among the intergenic SNVs used to estimate these rates, singletons and doubletons account for only ~25%^7^, so most variants occur at a higher frequency and thus likely arose hundreds or thousands of generations in the past. Over such a long time span, variants affected by cryptic selection, gBGC, or other evolutionary processes are more likely to have been fixed or disappeared, altering the distribution of observable variation.

Because ERVs are assumed to have occurred very recently in human history, we asked if ERV-based mutation rate estimates differed from the 1000G estimates, and if so, whether our revised estimation strategy more accurately represents basal mutation processes. To answer these questions, we first used the BRIDGES ERVs to estimate mutation rates according to mutation type (e.g., A > C, A > G, and so on) and local sequence context, considering the bases up to 3 positions upstream and downstream from each variant site (Methods). We refer to a mutation of a given type centered at a given sequence motif as a “mutation subtype” (e.g., C[A > C]G is a 3-mer subtype). Note that we are not estimating an absolute per-site, per generation mutation rate, but rather the relative fraction of each subtype containing an ERV within the BRIDGES data. We refer to rates calculated in this manner as “relative mutation rates,” and estimated these rates for all possible 1-, 3-, 5-, or 7-mer subtypes (Supplementary Data 1).

**Fig. 1:**
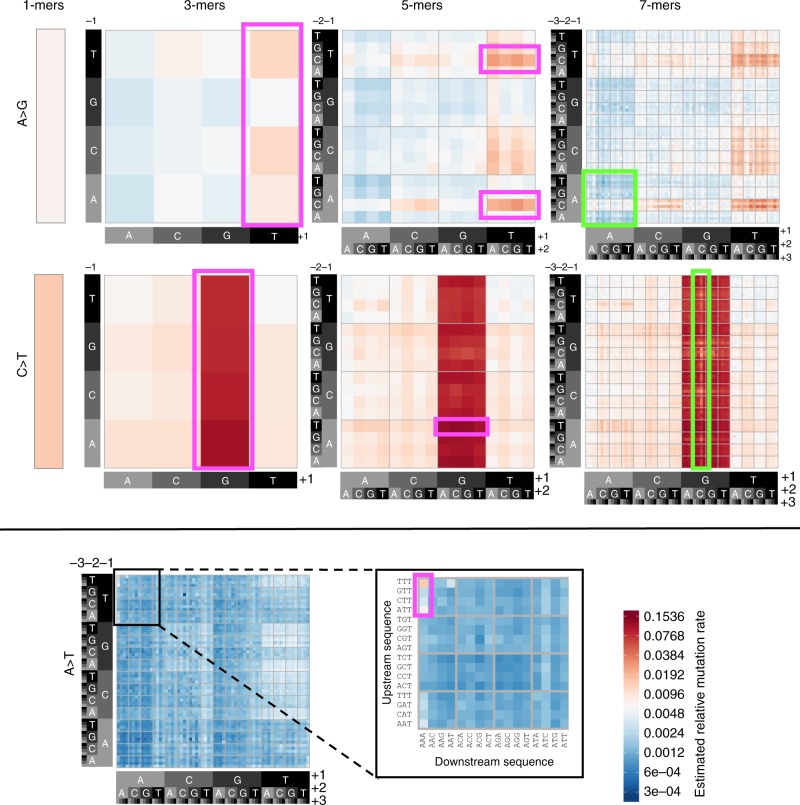
Mutation rates vary according to sequence context. **a** Heatmap of estimated relative mutation rates for all possible for A > G and C > T transition subtypes, up to a 7-mer resolution (high-resolution heatmaps for all possible subtypes are included in Supplementary Fig. 1). The leftmost panels show the relative mutation rates for the 1-mer types, and the subsequent panels to the right show these rates stratified by increasingly broader sequence context. Each 4 × 4 grid delineates a set of 16 subtypes, defined by the upstream sequence (*y*-axis) and downstream sequence (*x*-axis) from the central (mutated) nucleotide. Boxed regions indicate motifs previously identified by Aggarwala and Voight as hypermutable (pink) or hypomutable (green), relative to their similar subtypes. **b** Zoomed-in view showing hypermutable NTT[A > T]AAA subtypes relative to other 7-mer A > T subtypes

ERV-derived relative mutation rate estimates for the six basic 1-mer mutation types reflect the expected higher mutability for transitions relative to transversions^1^. Splitting each mutation type into more granular subtypes reveals how additional patterns of mutation rate heterogeneity emerge as broader sequence contexts are incorporated (Fig. 1; Supplementary Fig. 1). Our ERV-based estimates confirm nearly all of the hypomutable or hypermutable motifs previously reported by Aggarwala and Voight^7^ and Panchin et al.^13^. A subset of these are highlighted in Fig. 1a, including lower relative mutation rates for NNN[C > T]GCG subtypes and A > G subtypes in motifs containing runs of four or more A bases (shown in green boxes), and higher relative mutation rates for N[A > G]T, N[C > T]G, and CA[A > G]TN subtypes (pink boxes). Another notable example of context-dependent hypermutability is the set of NTT[A > T]AAA subtypes (Fig. 1b), also described previously^7^. Despite A > T mutations having the lowest relative mutation rate among 1-mer types, its NTT[A > T]AAA subtypes have a > sixfold higher rate than the 1-mer A > T relative mutation rate.

Overall, the ERV-derived 7-mer relative mutation rates span a >400-fold range from 0.0003 (CGT[A > T]CCG) to 0.1416 (ATA[C > T]GCA). For every 3-mer subtype, we found overwhelming evidence for heterogeneity in the relative mutation rates among their 16 respective 5-mer constituents (chi-squared tests; all *P* < 10^−231^). Further, 1522 (99%) of the 1536 5-mer subtypes had significantly heterogeneous rates among their respective 7-mer constituents (chi-squared tests; *P* < 0.05) (Methods).

### Mutation rate estimates differ between ERVs and common SNVs

**Fig. 2:**
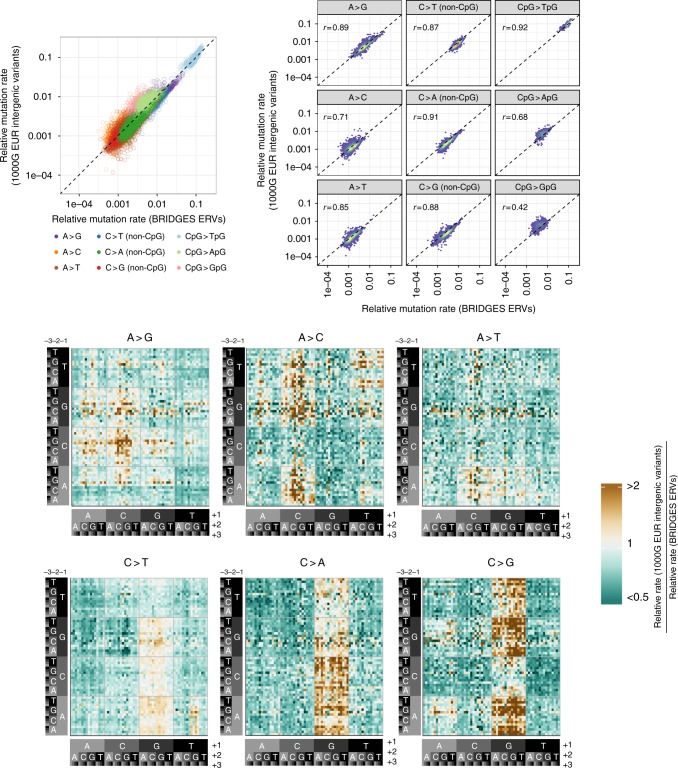
Discordance between ERV-estimated and common SNV-estimated mutation rates. **a** Relationship between 7-mer relative mutation rates estimated among BRIDGES ERVs (*x*-axis) and the 1000G intergenic SNVs (*y*-axis) on a log-log scale. We note that the strength of this correlation is driven by hypermutable CpG > TpG transitions. **b** Type-specific 2D-density plots, as situated in the scatterplot of **a**. The dashed line indicates the expected relationship if no bias is present. **c** Heatmap showing ratio between the relative mutation rates for each 7-mer mutation subtype. Subtypes with higher rates among the 1000G SNVs (relative to ERV-derived rates) are shaded gold, and subtypes with lower rates in the 1000G SNVs are shaded green. Relative differences are truncated at 2 and 0.5, as only 2.5% of subtypes showed differences beyond this range

We next compared the 7-mer relative mutation rates, estimated either from the BRIDGES ERVs or 1000G intergenic SNVs, to determine if our ERV-based estimates differ from previously reported patterns of mutation rate heterogeneity. Across all 24,576 7-mer mutation types, relative mutation rates were highly correlated between the two sets of estimates (Spearman’s *r* = 0.95; Fig. 2a). However, when stratified by mutation type, these correlations were often much weaker (*r* = 0.42–0.92; Fig. 2b). Considering differences in the estimated rates for each individual 7-mer subtype, we found 13% of 7-mer subtypes had differences of 50% or more between the two estimates after normalization. These discrepancies did not occur randomly across subtypes (Fig. 2c). For example, relative mutation rates for CpG > ApG and CpG > GpG transversions were, respectively 26% and 39% higher in the 1000G estimates compared to the ERV-derived estimates. Sequence context also affects relative mutation rate estimates for A > C and A > G subtypes: 1000G-derived estimates were significantly higher than ERV-derived estimates among GC-rich motifs (4–6 G/C bases in the ±3 bp flanking sequence) compared to low-GC motifs (three or fewer flanking G/C bases) (*t*- tests; *P* < 8.0 × 10^−30^) (Supplementary Fig. 2; Supplementary Table 2). This observation is consistent with the known correlation between GC content and biased gene conversion^18,31^, though other evolutionary processes may also have contributed.

We considered the possibility that these patterns of dissimilarity were simply due to technical differences between the BRIDGES and 1000G samples. To address this concern, we estimated 7-mer relative mutation rates using 12,088,037 variants with a minor allele count ≥10 (MAC10+) in the BRIDGES sample and compared these estimates to the ERV-derived and 1000G-derived estimates (Supplementary Note). Importantly, the MAC10+ 7-mer relative mutation rates were more closely correlated with the 1000G-derived estimates (overall: *r* = 0.98; Supplementary Fig. 3a; type-specific: *r* = 0.87–0.98; Supplementary Fig. 3b), than with the ERV-derived estimates (overall: *r* = 0.95; Supplementary Fig. 4a; type-specific: *r* = 0.45–0.95; Supplementary Fig. 4b). Like the 1000G estimates, the MAC10+ estimates also showed higher rates of CpG transversions and A > G/A > C mutations in GC-rich motifs (Supplementary Fig. 4c), but between the MAC10+ and 1000G estimates, these differences were absent or much weaker (Supplementary Fig. 3c).

Collectively, these results suggest that the dissimilarities between ERV-based and common SNV-based estimates are driven not by differences in the data source or analysis pipeline, but by differences in the allele frequencies of the variants used to estimate the rates. There are two plausible explanations for these differences: either (1) the ancestrally older variants included in the 1000G data are under the influence of evolutionary processes that have altered the relative frequencies among subtypes, or (2) even after our careful data cleaning and filtering, certain sequence motifs are enriched for false-positive or false negative sequencing errors in the BRIDGES ERVs.

These scenarios can be tested by comparing which set of estimates better describes the observed distribution of true de novo mutations. We reasoned that if biased sequencing errors have occurred, such spurious effects would occur more frequently among BRIDGES ERVs, as errors must be present in multiple individuals to manifest among the common variants included in the 1000G data. In such a scenario, we would expect the 1000G estimates to explain the distribution of true de novo mutations more accurately. In contrast, if the relative mutation rate estimates have been influenced by evolutionary processes, such biases should have a stronger effect on the 1000G estimates and the ERV-derived estimates would provide a better fit.

### ERVs accurately predict de novo mutations

We implemented this validation strategy by comparing how accurately different sets of relative mutation rate estimates predicted the incidence of 46,813 bona fide de novo mutations collected from two family-based WGS datasets: The Genomes of the Netherlands (GoNL) project^9^ and the Inova Translational Medicine Institute Preterm Birth Study^12^ (ITMI) (Methods; Supplementary Fig. 5). We set these de novo mutations against a randomly selected background of 1 million nonmutated sites, then applied logistic regression models using each set of relative mutation rate estimates (either ERV-based estimates at varying K-mer lengths, or 1000G-based 7-mer estimates) to predict the log-odds of observing a de novo mutation at each of the 1,046,813 sites. We evaluated model performance by two likelihood-based goodness-of-fit statistics: the Akaike information criterion (AIC), and Nagelkerke’s pseudo-*R*^2^ (Methods). Each model has one parameter, so the AIC of each model is −2log-likelihood+2.

Among ERV-based K-mer models, goodness-of-fit improved consistently with consideration for longer motifs, with the 7-mer model producing the best fit overall (Table 1). These trends did not change when varying the number of nonmutated sites (Supplementary Table 3) nor when applied exclusively to either the GoNL or ITMI mutations (Supplementary Table 4), indicating the regression was not merely fitting to cryptic errors in the validation data. To assess if our results are affected by mapping artifacts, we also re-estimated the ERV-based 7-mer relative mutation rates after applying the 1000 Genomes strict accessibility mask (Supplementary Note). The masked and unmasked 7-mer rates are highly concordant, and most discrepancies appear to be an artifact of sampling variation due to fewer ERVs in the masked data (Supplementary Fig. 6). When applied to predict the de novo mutations, the masked rates produced a worse fit than the unmasked rates (Table 1), suggesting that the reduction in ERVs caused by applying the mask has a larger effect on the precision of our estimates than any mapping artifacts present in the unmasked data. We next analyzed each mutation type separately to determine if the same trend of improved goodness-of-fit using longer K-mers held for different mutation types. In each of these type-specific validation models, the ERV-based 7-mer relative mutation rate estimates provided a significantly better fit than estimates in smaller K-mers (Supplementary Table 5).

**Table 1:** Goodness-of-fit statistics for mutation rate estimates applied to de novo testing data

Mutation rate estimation strategy			*AIC*	Δ*AIC*^a^	*AIC* rank^b^	Nagelkerke’s *R*^2^
Subtype length	Study	Variant type				
1-mers	BRIDGES	ERVs	353,896	21,575	7	0.088
3-mers	BRIDGES	ERVs	335,319	2998	4	0.118
5-mers	BRIDGES	ERVs	332,861	540	3	0.124
7-mers	BRIDGES	ERVs	332,321	0	1	0.126
7-mers	BRIDGES	ERVs (passing 1000G strict mask)	332,582	261	2	0.125
7-mers	BRIDGES	MAC10+	342,886	10,565	5	0.103
7-mers	1000G	Intergenic SNVs^7^	344,003	11,682	6	0.100

^a^Difference in AIC from the baseline BRIDGES 7-mer model

^b^Lower AIC rank indicates better model performance

We then compared the goodness-of-fit of the BRIDGES ERV-based K-mer models with the 7-mer model based on 1000G intergenic SNVs. Although Aggarwala and Voight^7^ demonstrate that the 1000G 7-mer model significantly improves on 5-mer or 3-mer models, our results show that all ERV-based models (except the 1-mer model) predict de novo mutations more accurately than 1000G 7-mer model (Table 1). Considering each mutation type separately (Supplementary Table 5), we find that the performance of the 1000G 7-mer model is particularly weak among certain mutation classes: for A > C and A > G types, the 1000G 7-mer models provide a worse fit than ERV-derived 5-mer models, and for A > T and CpG > GpG types the fit is worse than ERV-derived 3-mer models. In each of the other C > N types, the 1000G 7-mer model performs comparably to the ERV-derived 7-mer model, indicating the inferred mutation patterns of these types are mostly consistent between the two datasets. These results thus support a scenario where, due to the influence of gBGC^17^ or changing mutation processes^19^, type- and subtype-specific patterns of variation among the 1000G-derived estimates are less accurate than ERV-derived estimates in capturing ongoing patterns of germline mutability.

### Subtype-specific mutagenic effects of genomic features

Family-based sequencing studies have been instrumental in identifying genomic features that are associated with variation in the germline mutation rate^9,11,25^. However, these studies have only described the marginal effects of features on the entire spectrum of mutation, and have not assessed if the effect of a genomic feature might vary according to the local sequence context. To determine how the mutation distribution varies across the genomic landscape, we selected 14 genomic features (Supplementary Table 6) and estimated the joint effects of these features on the mutation rate of each 7-mer subtype using multiple logistic regression (Methods). Subtypes with few observed ERVs have little power to detect significant associations, so we estimated the effects of features only for the 24,396 of 24,576 (99.3%) 7-mer subtypes with at least 20 observed ERVs, resulting in 392,128 parameter estimates (Supplementary Data 2; Supplementary Fig. 7). We note that >84% of the 7-mer subtypes we evaluated contained >10 times as many ERVs as parameters estimated, so these estimates are unlikely to be an artifact of overfitting. To identify significant effects among the many associations tested, we applied a false discovery rate cutoff of 0.05 to the *P*- values for each feature across all subtype-specific estimates. Of the 24,396 7-mer subtypes analyzed, 3481 had at least one genomic feature significantly associated with mutability, with 6152 significant associations among 392,128 tests.

**Fig. 3:**
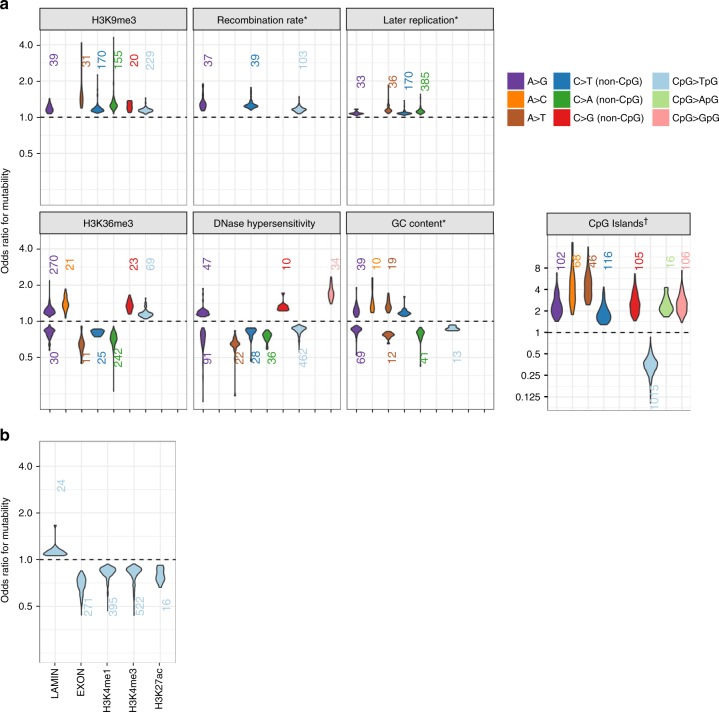
Distributions of statistically significant mutagenic effects of genomic features. **a** Effects of seven genomic features where associations with multiple mutation types were detected. For features with bidirectional effects, we separately plotted distributions of positive associations (OR > 1; above dashed line) and negative associations (OR < 1; below dashed line). The number of 7-mer subtypes within each type for which that feature is statistically significant in a positive or negative direction is shown above or below each distribution. Distributions are only shown for types with 10 or more 7-mer subtypes associated in the same direction. *Odds ratios for the three continuously valued features (recombination rate, replication timing, and GC content) indicate the change in odds of mutability per 10% increase in the value of that feature. ^†^Effects in CpG islands tend to be stronger than other features, so are shown on a wider scale. **b** Distributions of significant mutagenic effects for the 5 features only associated with CpG > TpG transitions

Three features (H3K9me3 peaks, recombination rate, and later replication timing) were associated with higher relative mutation rates across nearly all significantly associated 7-mer subtypes (Fig. 3a), consistent with previously reported mutagenic effects of these features: H3K9me3 marks are one of the strongest predictors of somatic SNV density^32,33^, and recombination and late replication timing are both correlated with higher germline mutation rates^8,9^. In addition, four features (H3K36me3 peaks, DNase hypersensitive sites [DHS], GC content, and CpG islands) were each associated with both higher and lower relative mutation rates, depending on the mutation type and, in some cases, the sequence motif. These features have been previously implicated in variation in germline or somatic mutation rates, but only as marginal effects, not type- or subtype-specific. H3K36me3 has been shown to regulate DNA repair machinery in vivo^34,35^. DNase hypersensitivity was previously reported to be associated with increased germline mutation rates^25^, though cancer genome studies have claimed DHS are susceptible to both increased and decreased somatic mutation rates^36,37^. CpG islands were associated with ~threefold lower mutation rates in 99% (1015/1024) of CpG > TpG 7-mer subtypes, consistent with known patterns of DNA hypomethylation in CpG islands^38^, but are associated with higher relative mutation rates in subtypes of other types.

Finally, for CpG > TpG transition subtypes, lamin-associated domains were associated with higher relative mutation rates and three histone marks (H3K4me1, H3K4me3, and H3K27ac) were associated with lower relative mutation rates (Fig. 3b). These results are consistent with published findings of correlations between these features and DNA methylation: lamin-associated domains were previously found to associate with focal DNA hypermethylation in colorectal cancer^39^, and H3K4me1, H3K4me3, and H3K27ac are known markers of DNA hypomethylation^40–42^. Exonic regions were associated with lower relative mutation rates for ~26% of CpG > TpG subtypes (Fig. 3b), consistent with findings of lower somatic SNV density in gene-rich regions^32^, though it is unclear if this is also due to DNA hypomethylation.

### Estimated effects of features predict de novo mutations

**Fig. 4:**
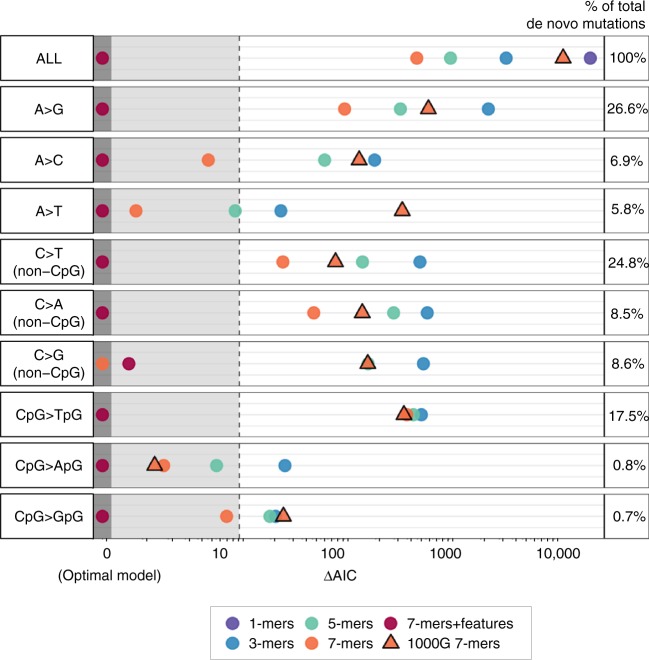
Comparison of goodness-of-fit for different mutation rate estimation strategies. For each mutation type and each model *i*, we calculated $\Delta {\mathrm{AIC}}_i = {\mathrm{AIC}}_i - {\mathrm{AIC}}_{\mathrm{min}}$ as a measure of relative model performance, with lower values of ΔAIC indicating better fit to the GoNL/ITMI de novo mutation data. ΔAIC is shown on the horizontal axis on an arcsinh scale. For each mutation type, the best-fitting model thus has a ΔAIC = 0. Models with ΔAIC < 10 (grey-shaded area) are considered comparable to the optimal model, whereas models with ΔAIC > 10 are considered to explain substantially less variation than the optimal model^67^

We applied these 7-mer + features mutation rate estimates to predict the GoNL/ITMI de novo mutations, using the same evaluation framework described earlier. Model fit statistics indicate that the rates estimated from 7-mer sequence context and genomic features describe the distribution of de novo mutations significantly better than the 7-mer-only estimates (Fig. 4). When partitioned by mutation type, inclusion of genomic features improves model fit for eight of the nine basic mutation types. These differences tend to be weaker among transversion types, likely because there were fewer de novo mutations of these types available (Fig. 4). Including genomic features had the largest effect on the prediction of CpG > TpG transitions, consistent with the expected associations between certain features and DNA methylation. Comparing the distribution of predicted mutations across basic types under different models, we find that all models generally recapitulate the observed distribution of de novo mutations, but the 1000G 7-mer model predicts a notably higher proportion of CpG > NpG mutations (Supplementary Fig. 8a). Stratifying by 3-mer subtype, the 1000G 7-mer predictions also tend to be more dissimilar from the de novo distribution than ERV-based 7-mer + features predictions (Supplementary Fig. 8b).

To further demonstrate that effects of genomic features described in Fig. 3 are supported by bona fide de novo mutation data, we pooled all subtypes found to be associated with each feature in a positive or negative direction and respectively tested for an enrichment or depletion of GoNL/ITMI de novo mutations in regions covered by that feature (Methods). We found 10 of the 20 tests were statistically significant in the expected direction (chi-squared tests; *P* < 0.05), confirming that, at a coarse level, many of the subtype-specific effects of genomic features inferred using ERVs are recapitulated among true de novo mutations (Supplementary Table 7).

### Germline mutation rates mirror somatic mutation processes

The rate heterogeneity between mutations of the same type suggests that distinct mutation mechanisms underlie some of the feature-subtype associations detected by our model. However, mechanisms for specific mutation signatures have mostly been studied for somatic mutations in cancer, and the degree to which these mechanisms affect germline mutations is generally unknown. In the following, we show two examples where the germline mutation rates from our data are consistent with mutation mechanisms observed in cancer. Moreover, we hypothesize a previously undescribed mechanism for germline point mutations.

In cancer genomes, H3K36me3-marked regions are targeted by the error-prone DNA polymerase eta (POLH, also known as pol *η*)^35^. Human POLH is particularly biased towards generating A > G mutations at sites flanked by weak (A or T, denoted as W) bases^43^; consequently, H3K36me3-marked regions are enriched for W[A > G]W mutations in various cancers^35^. In our data, among the 403 7-mer subtypes showing significant positive associations with H3K36me3-marked regions, a significant majority (270, or 67%) are A > G subtypes (exact binomial test; *P* < 1.09 × 10^−111^). Within the 270 positively associated A > G subtypes, 175 (65%) are W[A > G]W 3-mer subtypes, significantly more than expected by chance (exact binomial test; *P* < 4.12 × 10^−43^).Thus, our results suggest the H3K36me3-mediated POLH mutation signature also appears in the germline.

Active transcription factor binding sites (i.e., occurring in DHS) are also prone to elevated somatic mutation rates in various cancers, likely because bound transcription factors make DNA inaccessible to nucleotide excision repair (NER) machinery^37,44^. For example, the CCAAT motif is a highly specific binding target for the trimeric nuclear factor Y (NF-Y) complex^45^, and active NF-Y binding sites show a >3.2-fold enrichment for somatic mutations in melanomas^37^. Our results indicate that transcription factor binding may also explain motif-specific hypermutability in the germline. Among the 7-mer subtypes positively associated with DHS, CCA[A > G]TNN subtypes show a 1.1–1.3-fold enrichment (Wald test; *P* < 2 × 10^−4^), and the CCA[A > G]TNN de novo mutation rate in the GoNL/ITMI dataset is 1.7-fold higher when occurring within DHS versus non-DHS regions (1-df chi-squared test; *P* < 0.0055).

Finally, we and others^7^ observed that NTT[A > T]AAA subtypes have >sixfold higher mutation rates than other A > T subtypes (Fig. 1b). We note that the TTAAAA hexamer is the canonical insertion target for Long Interspersed Element 1 (LINE-1, or L1) retrotransposons, and is nicked by the L1-encoded ORF2p endonuclease at the antisense 3′-ApT-5′ dinucleotide^46^. These nicks produce T-rich 3′ flap structures, which can be recognized and removed by NER machinery, inhibiting L1 insertional mutagenesis, but leaving an A-rich single-strand break^47^. In transcriptionally active regions of the genome, such lesions are usually repaired by high-fidelity NER pathways^48^, but in nucleosomal DNA, where NER activity is impaired, the lesions are likely bypassed by error-prone translesion synthesis (TLS) polymerases^37^. Our results show NTT[A > T]AAA mutations are reduced >threefold when occurring in DHS (Wald test; *P* < 2.0 × 10^−26^). We hypothesize that the context-dependent mutation signature in our data is the result of damage induced by L1 retrotransposons and subsequent errors of the TLS polymerase. This model is consistent with observing higher NTT[A > T]AAA mutation rate outside of DHS, where NER activity may be impaired and lesions must be bypassed by error-prone TLS during replication. Additionally, according to the “A-rule”^49^, TLS polymerases preferentially pair abasic sites with adenine. Hence, mutations generated by errors of the TLS polymerase explain the preponderance of A > T (but not A > G or A > C) mutations at the NTTAAAA motif.

## Discussion

The main motivation of our study is to understand the genome-wide variation of germline mutation rates in humans. We bring to this task two innovations: first, we take advantage of large-scale WGS data, focusing on ERVs as a potentially more powerful data source than currently available collections of de novo mutations^9,10,12,25^ or common variants^7,13^. Second, building upon previous attempts to holistically model the relationship between sequence context, genomic features, and mutation rate, we estimate fine-scale mutagenic effects of multiple genomic features. Unlike previous studies, which estimated the impact of genomic features by treating all single-nucleotide mutation subtypes in aggregate^25^, we allow for the possibility that mutation rates of sequence motifs are differentially affected by these features.

Our results not only confirm the previously reported hypermutable effects of specific sequence contexts and genomic features, but also demonstrate that many feature-associated effects previously only described in somatic cells are present in the germline. Moreover, our approach identifies certain genomic features, including H3K36me3 peaks, DNase hypersensitive sites, and CpG islands, that may act to both suppress and promote mutability depending on the mutation type and sequence context, providing insight into the causal mechanisms of germline mutation rate heterogeneity across the genomic landscape.

The subtype-specific effects of genomic features we report likely represent only a fraction of the effects across the genome, due to the limited power of detecting associations among rarer subtypes. A larger dataset of ERVs will likely reveal additional cases of association and will enable further study of mutation patterns among longer sequence motifs, additional genomic features, and interactions or nonlinear effects thereof. We also note several of the genomic features used in our study were assayed in somatic cell lines or aggregated over multiple cell types. The currently available data for these features only crudely approximates the true genomic variation in germ cells, so the effects we estimated have likely regressed toward the mean. Generating precise maps of genomic features within male and female germ cell lineages may further uncover mutagenic mechanisms unique to the germline. Despite these limitations, the fine-scale effects of sequence context and genomic features reported here provide the most accurate map to date of germline mutation variation, as demonstrated by their improved ability to predict genuine de novo mutation patterns.

Even without accounting for the effects of genomic features, our ERV-derived mutation rate estimates for 7-mer subtypes are consistently more accurate than those based on mostly common SNVs from 1000 Genomes Project data^7^. Remarkably, even coarser estimates—the ERV-derived 5-mer and 3-mer rates—predict the spectrum of de novo mutations more accurately than the 1000G 7-mer estimates, demonstrating the merit of ERVs as a refined data resource for studying innate mutation patterns. Some of the improvement is likely the result of reduced sampling error, as our ERV dataset is larger than the 1000G dataset. Nevertheless, this result has two important implications. First, it suggests that high-frequency variants in presumably neutral genomic regions are influenced by biased evolutionary processes, such as selection and gBGC, or these variants arose via past mutational processes that are now inactive^19^. Second, this reaffirms the high quality of ERVs in our data: the potential errors due to calling or mapping biases among these ERVs are likely weaker than the evolution-driven biases affecting the older variants. The larger sample, young allelic age, and high quality of ERVs together result in a demonstrably more accurate appraisal of recent or ongoing patterns of mutability than common SNVs.

Because the germline mutation rate is a critical parameter in the study of genetic variation, we envision a wide range of applications that stand to benefit from incorporating our genome-wide map of mutation rate estimates. Currently, many methods that rely on simulating “baseline” mutations, such as the pathogenicity scoring algorithm *CADD*^50^ and coalescent simulator *ms*^51^, do not account for context-dependent mutation rate differences. Likewise, clinical applications for differentiating disease-causing mutations from background variation require a precise estimate of the expected de novo mutation rate, but even the most advanced of these only consider differences in 3-mer or 7-mer sequence contexts, and are based on intergenic SNVs from 1000 Genomes data^7,52^. Incorporating more accurate sequence- and feature-dependent estimates of mutation rates may lead to more realistic simulations and greater confidence in the inferences made by these methods. Another relevant area of research where our results might be applicable is the study of how germline mutation mechanisms have evolved over time^19,53,54^. If mutator phenotypes have frequently arisen throughout the evolutionary history of humans (as hypothesized by Harris and Pritchard^19^), the effects of mutational modifiers have likely been extremely subtle, manifesting as granular context-specific mutation signatures. Our results, which describe the present-day pattern of mutation rate heterogeneity in Europeans, provide a wealth of potential hypotheses for investigating how these mutation processes have been shaped via past evolution.

To facilitate the use of our genome-wide mutation rate estimates in other analysis and simulation pipelines, we have created a genome browser track to visualize these estimates at a single-base resolution alongside other genomic data. Ultimately, the refined mutation patterns from ERVs and the detailed dissection of context-feature effects serves as a quantitative foundation for better understanding the molecular origins of mutation rate heterogeneity and its consequences in heritable diseases and human evolution.

## Methods

### Sample description

The BRIDGES sample contains 3927 unrelated European American bipolar disorder cases and controls. The cases and controls from the Centre for Addiction and Mental Health (CAMH) in Toronto (*n* = 830), the Institute of Psychiatry, Psychology and Neuroscience (IoPPN) and King’s College London in London, UK (*n* = 845)^55^, the Genomic Psychiatry Cohort (GPC) (*n* = 1151)^56^, and the Prechter Repository (*n* = 363)^57^ were collected as previously described, as were the STEP-BD cases (*n* = 304), obtained from the NIMH repository^58^, and the Minnesota Center for Twin and Family Research (MCTFR) study controls (*n* = 434)^59^. In all studies, DNA was extracted from blood-based samples. All human research was approved by the relevant institutional review boards and conducted according to the Declaration of Helsinki. All participants provided written informed consent.

### Sample library preparation

The concentration of each DNA sample was measured by fluorometric means (PicoGreen, Thermo Fisher, Woburn, MA, USA) followed by agarose gel electrophoresis to verify the integrity of DNA. Six-hundred nanograms of DNA was sheared with acoustic shearing (Covaris, Woburn, MA, USA) to an average size of 400 nt. Following shearing, the samples are transformed to a sequencing library using standard protocols to create a paired-end library. Briefly, sheared DNA was end-repaired, A-tailed and ligated with Illumina adaptors (New England Biolabs, Ipswitch, MA, USA). Following ligation, indexed primers were used to amplify the final libraries for each sample. Each sample received two indexes: 96 i7 indexes were used to identify each sample in each 96-well reaction plate while a single i5 index was used for each plate. This combination of indexes uniquely coded all samples in the project when both the i7 and i5 indexes were read during sequencing. Following six cycles of PCR (Kapa Biosystems, Wilmington, MA, USA), libraries were purified and quality controlled by assaying the final library size using the Agilent Bioanalyzer (Agilent Technologies, Santa Clara, CA, USA) and quantitating the final library via real-time PCR (Kappa Biosciences). A single peak between 300 and 400 bp indicates a properly constructed and amplified library ready for sequencing. PCR cycles for amplification are kept to a minimum to minimize PCR duplication rate and maximize library complexity.

### Sequencing

Sequencing was performed per Illumina protocol, essentially as described by Bentley et al.^41^. Libraries were pooled in sets of 12 samples and each pool sequenced on a single lane of a HiSeq 2500 flowcell using version 3 Illumina chemistry at paired-end 100 nt read lengths. Each library pool was loaded at 13 pM to generate 160–180 M paired reads per lane. Multiple flowcells of the library pools were performed to generate a final data set with an average coverage of 9.6× per sample.

### Sample filtering and data quality control

Among the 3927 samples attempted, three failed library preparation and were not sequenced. We removed an additional 162 samples due to quality issues: five with imbalanced read counts between read 1 and read 2, four with improperly generated BAM files, 16 that had an average coverage <3×, and 137 due to high contamination (FREEMIX or CHIPMIX score >3% using VerifyBAMID^60^). For samples that failed for multiple reasons, we report a single category for simplicity.

Among these 3762 samples, reads were mapped to Build 37 of the human reference genome (including decoy sequence^28^), with alignment and variant calling performed using the GotCloud pipeline^61^. After variant calling, we applied additional sample-level filtering as described below to obtain the 3716 included in our analysis. We first excluded 10 case samples that were not phenotyped as type 1 bipolar disorder (removed solely for consistency with ongoing analyses of the BRIDGES data that do require phenotypes). We identified and removed an additional 23 samples that showed evidence of sample swaps in VerifyBAMID^60^, but had not been excluded from variant calling. We next computed continental-ancestry PCA coordinates by projecting BRIDGES samples in the coordinate space of the 1000 Genomes phase 1 samples^62^. We dropped 11 samples identified as PC ancestry outliers, defined by PC1 < 0.01 or PC2 < 0.025. We then checked for relatedness using the $\hat \pi$ statistic (i.e., estimation of pairwise identity-by-descent based on LD-pruned SNPs), computed in plink^63^. Nearly all pairwise sample comparisons were consistent with being unrelated, with $\hat \pi < 0.05$ for 99.9% of sample pairs. Two samples were dropped due to relatedness, as the $\hat \pi$ between these was 0.5, indicating the two were full siblings.

These filters reduced the sample to 3716 individuals, in which we called 37,470,516 autosomal singleton SNVs in the mappable genome (i.e., non-N reference bases in the GRCh37 reference genome) that passed the variant-level filtering criteria implemented in the GotCloud pipeline^61^. Prior to performing our analyses, we examined how these 37.5 million ERVs were distributed across individual samples to identify and remove individuals that showed abnormal patterns of variation due to systematic sequencing errors or batch effects. In brief, we adapted the nonnegative matrix factorization (NMF) technique described by Lawrence et al.^64^ to summarize the distribution of ERVs unique to each individual as a composite of three distinct “signatures.” For each of the 3716 individuals in our sample, we calculated a vector of 96 3-mer relative mutation rates (described below) using only the ERVs observed in that individual, generating a 3716 × 96 rate matrix. Decomposition of this matrix via NMF produces a 3716 × 3 matrix describing the relative contribution of each signature to the observed mutation spectrum per individual. Because we assume the relative mutation rate of any given subtype should be similar across individuals, it follows that the contribution of a given NMF signature should also be similar. We removed 156 individuals where one or more signatures had a contribution >2 standard deviations away from the mean contribution of that signature calculated across all individuals, reasoning that ERVs observed in these individuals are more likely to be errors. The final sample used in our analyses thus consists of 3560 individuals, in which we identified 35,574,417 singletons. Additional details of this filtering strategy are described in the Supplementary Note.

### Mutation subtypes and calculation of relative mutation rates

Each of the 35,574,417 singletons can be classified into one of 6 basic mutation types, defined by the reference and alternative allele: A > C, A > G, A > T, C > T, C > G, and C > A. The notation of A > C includes both A-to-C mutations and complementary T-to-G mutations. For each mutation type, we further define a set of mutation subtypes by the bases flanking the variant site. Since there are 4 possible bases at both the +1 position and the −1 position, there are 4 × 4 = 16 possible 3-mers containing each basic mutation type at the central position, producing 6 × 16 = 96 3-mer subtypes. Likewise, there are 6 × 4^4^ = 1536 5-mer subtypes, and 6 × 4^6^ = 24,576 7-mer subtypes. To simplify notation, we denote a subtype by the sequence motif containing either an A or a C as the reference base at the central position (e.g., either CGT[A > X]TCG or CGT[C > X]TCG).

For each K-mer subtype, we divided the number of ERVs observed at the central position of the K-mer by the number of times the K-mer is seen in the mappable autosomal regions of the reference genome; we term this proportion the estimated relative mutation rate. K-mers in the reference genome were counted by a 1-bp sliding window, so that every possible occurrence of that K-mer was accounted for (e.g., a run of 4 As is counted as two AAA 3-mers shifted by one base). For example, we observed 7548 C >T or G > A autosomal singletons occurring in an ATACGCA or TGCGTAT 7-mer motif (the underlined base indicates the variant site) and there are 53,314 such motifs in the autosomal reference genome where this subtype of mutation could be observed, yielding a relative mutation rate estimate of 7548/53,314 = 0.1416 for the ATA[C > T]GCA subtype.

### Testing for heterogeneity of relative rates

As each K-mer can be split into 16 possible (K + 2)-mers that share the same internal motif but differ in their terminal bases, the relative mutation rate for each K-mer subtype is the weighted mean of the rates found among its 16 possible (K + 2)-mer constituent subtypes. To assess the heterogeneity of relative mutation rates among each set of 16 (K + 2)-bp constituent subtypes that share the same K-bp motif, we performed a chi-squared test for uniformity of these rates, with each test having 15 degrees of freedom.

### Mutation prediction model and validation

To evaluate the accuracy of different mutation rate estimation strategies, we applied the estimated rates to predict the incidence of 46,813 de novo mutations using logistic regression. These de novo mutations were published by two independent studies: 11,020 de novo mutations detected in 258 Dutch families by the GoNL project^9^, and 35,793 de novo mutations from 816 families sequenced by the ITMI Premature Birth Study^12^. We combined the observed mutations with 1 million randomly selected sites from the mappable autosomal regions of the reference genome to serve as a nonmutated background, reasoning that ~20 nonmutated sites for each actual de novo mutation would be sufficient to minimize sampling noise in the set of nonmutated sites; we also repeated this procedure with 500,000, 2 million, and 3 million randomly selected sites to tell if the trends we observed were affected by the size of the nonmutated background. Because each nonmutated site can be ambiguously considered as the background for three different mutation types, we divided the 1 million nonmutated sites into three nonoverlapping sets. We designated A/T and C/G reference bases in the first set (consisting of 333,334 unique sites) as nonmutated A > G and C > T types, respectively, and so on for the second set (A > C or C > G types), and the third set (A > T or C > A types), each of which contained 333,333 unique sites. Hence, we considered a total of 1,046,813 testing sites (1,000,000 unmutated sites and 46,813 de novo mutations), each with one possible mutation event, in our prediction models.

Now let ${{i}} = \left\{ {{{1}}, \ldots ,{{1046813}}} \right\}$ be an index for the 1,046,813 testing sites. We coded *d*_*i*_ = 1 if site ***i*** is a de novo mutation and *d*_*i*_ = 0 otherwise. If a set of estimated relative mutation rates reflects the underlying mutation process, we expect that the odds of a given site for carrying a de novo mutation increases with the estimated relative mutation rate of that site. To assess this expectation for all sets of mutation rate estimation strategies (e.g., ERV-based or 1000G-based 7-mer estimates), we annotated each testing site *i* with the relative mutation rate estimated under strategy *M* (*r*_*i,M*_), and used logistic regression to model the probability of a de novo mutation at each site as a function of these rate estimates, where *α*_0_ is the intercept term and *α*_1_ is the regression coefficient:

1 $${{ln}}\left( {\frac{{{{{Pr}}}\left( {{{d}}_{{i}} = {{1}}} \right)}}{{{{{Pr}}}\left( {{{d}}_{{i}} = {{0}}} \right)}}} \right) = {{\alpha }}_{{0}} + {{\alpha }}_{{1}}{{r}}_{{{i}},{{M}}}$$

The probability of a mutation at each testing site can then be calculated as:

2 $${{{Pr}}}\left( {{{d}}_{{i}} = {{1}}} \right) = \frac{{{1}}}{{{{1}} + {{{e}}}^{{{\alpha }}_{{0}} + {{\alpha }}_{{1}}{{r}}_{{{i}},{{M}}}}}}$$

The overall likelihood of model *M*, given the observed data, is the product of the probability values over all 1,046,813 sites:

3 $${{L}}_{{M}} = \mathop {\prod }\limits_{{{d}}_{{i}} = {{1}}} \frac{{{1}}}{{{{1}} + {{{e}}}^{{{\alpha }}_{{0}} + {{\alpha }}_{{1}}{{r}}_{{{i}},{{M}}}}}}\mathop {\prod }\limits_{{{d}}_{{i}} = {{0}}} \frac{{{{{e}}}^{{{\alpha }}_{{0}} + {{\alpha }}_{{1}}{{r}}_{{{i}},{{M}}}}}}{{{{1}} + {{{e}}}^{{{\alpha }}_{{0}} + {{\alpha }}_{{1}}{{r}}_{{{i}},{{M}}}}}}$$

Using this likelihood, we evaluated model fit by the Akaike Information Content (AIC), where *p* is the number of parameters in Eq. (1) (because all models are based on a single covariate of mutation rates, *p* = 1 in all cases):

4 $${{{AIC}}}_{{M}} = {{2}}{{p}} - {{2}}\ln \left( {{{L}}_{{M}}} \right)$$

For each model, we also calculate Nagelkerke’s *R*^2^:

5 $${{R}}_{{M}}^{{2}} = \frac{{{{1}} - \left\{ {\frac{{{{L}}_{{0}}}}{{{{L}}_{{M}}}}} \right\}^{{{2}}/{{N}}}}}{{{{1}} - \left\{ {{{L}}_{{0}}} \right\}^{{{2}}/{{N}}}}}$$

Here, *L*_0_ is the likelihood of a null intercept-only model with no covariates.

Because these likelihood-based goodness-of-fit statistics are calculated across all the basic mutation types combined, they do not provide information about which types benefit most strongly from using expanded sequence motifs. For example, it is possible that any improvement to the overall goodness-of-fit is elicited by context-dependent heterogeneity of a single mutation type, whereas other types might not be significantly affected by using longer sequence motifs, and do not contribute to the improved model fit. To identify these type-specific trends, we stratified our testing data by each of the basic mutation types. To account for the known hypermutability of cytosine at CpG dinculeotides, we separated C > T, C > G, and C > A mutations into CpG and non-CpG types, for a total of 9 basic mutation types. For each type, we repeated the 3-mer, 5-mer, and 7-mer models on only the sites of that type. Within each set of type-specific models, we again compared the goodness-of-fit using AIC and Nagelkerke’s *R*^2^. Note that because the absolute values of AIC and Nagelkerke’s *R*^2^ are a function of the number of data points included in the model, these statistics cannot be directly compared between type-specific models, where the number of data points vary.

### Estimating effects of local genomic features

We estimated the effect of 14 genomic features (data sources for these features are described in Supplementary Table 6) on the relative mutation rate of each 7-mer subtype using the following logistic regression framework. Let *K* be the index across all 7-mer subtypes with 20 or more observed singletons $\left( {{{K}} \in \left\{ {{{1}}, \ldots ,{{24396}}} \right\}} \right)$. Let ***j***_***k***_ be the index across all sites that are centered at the 7-mer motif that could produce a mutation of subtype *K*, and let ${{Z}}_{{{j}}_{{K}}} = {{1}}$ if the site carries a singleton of subtype *K* and ${{Z}}_{{{j}}_{{K}}} = {{0}}$ otherwise. We annotated each site of the considered subtype for 14 genomic features, generating predictors ${{F}}_{{{j}}_{{K}},{{1}}}, \ldots ,{{F}}_{{{j}}_{{K}},{{14}}}.$ We treated 11 of these features as binary variables (seven histone marks, lamin-associated domains, CpG islands, DNase hypersensitive sites, exons), setting the predictor ${{F}}_{{{j}}_{{K}},{{g}}} = {{1}},{{g}} \in \left\{ {{{1}}, \ldots ,{{11}}} \right\}$ if the central site of the motif was inside the specified regions and ${{F}}_{{{j}}_{{K}},{{g}}} = {{0}}$ otherwise. For the 3 continuous features (recombination rate, replication timing, surrounding GC content), we set the predictor ${{F}}_{{{j}}_{{K}},{{g}}},{{g}} \in \left\{ {{{12}},{{13}},{{14}}} \right\}$ to the mean value of that feature in a 10 kb window centered at the site. Because the inferred effect of some features may be confounded by correlation with read depth and calling rates (e.g., GC content^65^), we included read depth at the central site of the 7-mer as covariate ${{F}}_{{{j}}_{{K}},{{DP}}}.$ For each 7-mer subtype *K*, we then evaluated the effect of the genomic predictors on the log odds of mutability for each site ${{Z}}_{{{j}}_{{K}}}$ using the following logistic regression equation:

6 $${{{ln}}}\left( {\frac{{{{{Pr}}}\left( {{{Z}}_{{{j}}_{{K}}} = {{1}}} \right)}}{{{{{Pr}}}\left( {{{Z}}_{{{j}}_{{K}}} = {{0}}} \right)}}} \right) = {{\beta }}_{{0}}^{{K}} + {{\beta }}_{{1}}^{{K}}{{F}}_{{{j}}_{{K}},{{1}}} + \ldots + {{\beta }}_{{{14}}}^{{K}}{{F}}_{{{j}}_{{K}},{{14}}} + {{\beta }}_{{{DP}}}^{{K}}{{F}}_{{{j}}_{{K}},{{DP}}}$$

where $\left( {{{\beta }}_{{1}}^{{K}}, \ldots ,{{\beta }}_{{{14}}}^{{K}}} \right)$ are effects of the 14 considered genomic features on the mutation rate of subtype *K*, and ${{\beta }}_{{{DP}}}^{{K}}$ is the effect of the local sequencing depth. The intercept of this model, ${{\beta }}_{{0}}^{{K}}$, represents the feature-adjusted relative mutation rate for the considered 7-mer subtype. We performed this logistic regression and obtained parameter estimates in R v3.2.3 using the speedglm() function from the speedglm package. We performed this procedure for each of the ${{K}} \in \left\{ {{{1}}, \ldots ,{{24396}}} \right\}$ 7-mer subtypes; the resulting beta values and standard errors for 16 × 24,396 estimated parameters are provided in Supplementary Data 2. Note that we did not consider estimating interaction effects between the 14 genomic features, as estimating all 2-way interactions would require an additional 14*(13–1)/2 = 91 parameters per subtype-specific regression, which would lead to overfitting concerns.

To generate a map of mutation rates across the genome, we used the estimated regression coefficients to predict the relative mutation rate (i.e., probability of observing a singleton) at each site ***j*** where a mutation of a given 7-mer subtype could occur:

7 $${{{Pr}}}\left( {{{Z}}_{{{j}}_{{K}}} = {{1}}} \right) = \frac{{{{{exp}}}\left( {{{\beta }}_{{0}}^{{K}} + {{\beta }}_{{1}}^{{K}}{{F}}_{{{j}}_{{K}},{{1}}} + \ldots + {{\beta }}_{{{14}}}^{{K}}{{F}}_{{{j}}_{{K}},{{14}}} + {{\beta }}_{{{DP}}}^{{K}}{{F}}_{{{j}}_{{K}},{{DP}}}} \right)}}{{{{1}} + {{{exp}}}\left( {{{\beta }}_{{0}}^{{K}} + {{\beta }}_{{1}}^{{K}}{{F}}_{{{j}}_{{K}},{{1}}} + \ldots + {{\beta }}_{{{14}}}^{{K}}{{F}}_{{{j}}_{{K}},{{14}}} + {{\beta }}_{{{DP}}}^{{K}}{{F}}_{{{j}}_{{K}},{{DP}}}} \right)}}$$

Because there are three possible mutations at every site, we predict three independent mutation probabilities (one for each possible alternative allele). For example, for a site centered at a ACGATTG motif, we predict probabilities for A > C, A > G, and A > T alleles, using the parameters estimated from those models. This prediction uses all estimated effects, not just the effects determined to be statistically significant. We note that we did not generate predictions for sites within 5 Mb of the start/end of a chromosome, because recombination rate data were not available for these regions^66^.

To assess if inclusion of these genomic features improved upon the 7-mer mutation rate estimates in describing the true distribution of germline mutability, we again tested this model’s ability to predict the known de novo mutations from the GoNL^9^ and ITMI^12^ studies. We annotated each of the ${{i}} = \left\{ {{{1}}, \ldots ,{{1046813}}} \right\}$ testing sites with the predicted mutation rate, ${{{Pr}}}({{{Z}}_{{{i}}_{{K}}} = {{1}}})$, and calculated the goodness-of-fit using equations 1–5 with this parameter as the predictor. Note that the GoNL/ITMI data included de novo mutations within the 5 Mb telomeric regions where we could not estimate effects of genomic features. Rather than excluding sites in these regions from our goodness-of-fit comparison, we simply assigned the marginal 7-mer relative mutation rate as the predicted value for these sites, to ensure models were compared using identical data.

### Code availability

All custom scripts used in downstream data processing and analyses are available at https://github.com/carjed/smaug-genetics. A web-based utility and command-line code for annotating a variant call format (VCF) file of genetic variants with estimated 7-mer mutation rates can be accessed at http://www.jedidiahcarlson.com/mr-eel/.

## Electronic supplementary material

Description of Additional Supplementary Files

Supplementary Information

Supplementary Data 1

Supplementary Data 2

## Data Availability

We are in the process of submitting the BRIDGES sequence-based genotypes to dbGaP. K-mer-based relative mutation rate estimates are provided in Supplementary Data 1. The complete input data for our logistic regression models, containing feature annotations for the singletons and non-singletons of each 7-mer motif, are available at https://zenodo.org/record/1296396, and the parameter estimates are provided in Supplementary Data 2. Predicted mutation rates based on sequence context and genomic features at each site have been formatted as a UCSC Genome Browser track, which can be accessed at http://mutation.sph.umich.edu. All additional data generated and analyzed in this study are available from the authors upon request.
