# Supplementary material for: Extremely rare variants reveal patterns of germline mutation rate heterogeneity in humans
Source: Nat Commun. 2018 Sep 14;9:3753. doi: 10.1038/s41467-018-05936-5 (PMC6138700; doi:10.1038/s41467-018-05936-5)
Supplement: Supplementary file 1 — Description of Additional Supplementary Files [file 41467_2018_5936_MOESM1_ESM.pdf]

Description of Supplementary Data 1 and 2

Supplementary Data 1 Relative mutation rate estimates for 1-mers, 3-mers, 5-mers, and 7-mers

Each table contains data used to calculate relative mutation rates for K-mers of a given length. Each row in the table contains the following columns: 1) basic mutation type; 2) K-mer motif corresponding to a reference base A or C at the central mutated position (the reverse complement of each motif, corresponding to reference base T or G is given in parentheses); 3) number of singletons observed in the BRIDGES data of the K-mer subtype defined by columns 1 and 2; 4) total number of times the motif in column 2 is observed in the reference genome; 5) relative mutation rate of singletons in that subtype (column 3 divided by column 4). For 7-mer subtypes (Supplementary Table 2d), we include five additional columns: 6) number of singletons in that subtype that pass the 1000G strict accessibility mask; 7) number of motifs of that subtype that pass the 1000G strict accessibility mask; 8) relative mutation rate of the masked data (column 6 divided by column 7); 9) number of MAC10+ variants observed in the BRIDGES data of that subtype; 10) relative mutation rate of MAC10+ variants of that subtype (column 9 divided by column 4).

Supplementary Data 2 Parameter estimates for genomic features model

This table contains effect size estimates and standard errors of 16 parameters (14 features, plus intercept and read depth) for each of the 24,396 7-mer subtypes with at least 20 singletons in the BRIDGES data.
