# Supplementary material for: Extremely rare variants reveal patterns of germline mutation rate heterogeneity in humans
Source: Nat Commun. 2018 Sep 14;9:3753. doi: 10.1038/s41467-018-05936-5 (PMC6138700; doi:10.1038/s41467-018-05936-5)
Supplement: Supplementary file 2 — Supplementary Information [file 41467_2018_5936_MOESM2_ESM.pdf]

Supplementary information for "Extremely rare variants reveal patterns of germline mutation rate heterogeneity in humans" by Carlson et al.

Table of Contents

Supplementary Note ... 3
- Supplementary Note 1. Identification of outlier samples ... 3
- Supplementary Note 2. Estimation of false discovery rate by Ts/Tv statistics ... 4
- Supplementary Note 3. Potential sources of bias among ERVs ... 4
  - 3.1. Motif-specific error rates ... 4
  - 3.2. Mapping error ... 5
  - 3.3. Mispolarization of ERVs ... 6
- Supplementary Note 4. Curation of MAC10+-derived mutation rate estimates ... 7
- Acknowledgements ... 9
- Supplementary References ... 10

Supplementary Figures ... 11
- Supplementary Figure 1 ... 12
- Supplementary Figure 2 ... 13
- Supplementary Figure 3 ... 14
- Supplementary Figure 4 ... 15
- Supplementary Figure 5 ... 16
- Supplementary Figure 6 ... 17
- Supplementary Figure 7 ... 18
- Supplementary Figure 8 ... 19

Supplementary Tables ... 20
- Supplementary Table 1 ... 20
- Supplementary Table 2 ... 20
- Supplementary Table 3 ... 21
- Supplementary Table 4 ... 21
- Supplementary Table 5 ... 22
- Supplementary Table 6 ... 24
- Supplementary Table 7 ... 25

Supplementary Note

Supplementary Note 1. Identification of outlier samples

For the 3,716 individuals that passed our initial sample-level filters, we summarized the per-sample distribution of extremely rare variants (ERVs) across 3-mer subtypes and used this information to flag individuals that showed abnormal patterns of variation indicative of systematic sequencing errors or batch effects. In brief, we adapted the non-negative matrix factorization (NMF) technique described by Lawrence et al.[1] to deconvolute the 3-mer mutation spectra as a composite of 3 distinct "signatures." Assuming the population has been susceptible to the same mutation processes over the timespan in which ERVs have accumulated, we expect that the relative contribution of the 3 NMF signatures is stable across individuals. Applying this strategy, we identified 156 individuals where one or more signatures had a contribution >2 standard deviations away from the mean contribution of that signature (calculated across all individuals).

These outliers exhibited one of two distinct signatures indicative of error biases. The first signature, characterized by an unusually high proportion of C>A and G>T singletons, was overrepresented in 112 of these samples, consistent with patterns of oxidative damage that are known to occur during DNA shearing, likely due to the presence of reactive contaminants[2]. The second signature, characterized by depleted rates of C>N and G>N ERVs, was overrepresented in the remaining 44 samples. Further investigation of the samples carrying this signature showed many had higher GC bias scores (i.e., systematically lower depth of coverage in GC-rich regions), likely resulting in lower calling rates for C>N and G>N types. Moreover, 24 of the 44 samples were sequenced in the same batch, and the remaining 20 samples were distributed across only 8 of the 48 other batches, indicating that these coverage biases and resulting error signatures clustered by batch. To limit the confounding effects of nonbiological variation present in the data, we excluded the 156 samples displaying either of these error signatures. Note that doubletons in the pre-filtered sample that would have become singletons in the post-filtered sample were not included in our analysis. Many of these variants are likely true doubletons in the BRIDGES sample and hence present in the population at a higher frequency (i.e.,

having arose further in the past) than the average singleton, so retaining these ambiguous variants might inadvertently affect the distribution of variants.

Supplementary Note 2. Estimation of false discovery rate by Ts/Tv statistics

We estimate the false discovery rate among BRIDGES ERVs using the following method.

(1) Let $TS_o = TS_t + TS_f$ be the number of observed transitions (23,733,766), consisting of both true positives (TS_t), and false positives (TS_f)

(2) Let $TV_o = TV_t + TV_f$ be the number of observed transversions (11,840,651).

(3) Based on findings from other large-scale sequencing studies, the true positive Ts/Tv ratio, $TSTV_T = \frac{TS_t}{TV_t}$ is expected to be between 2.0 and 2.1[3].

(4) Because there are 8 possible transversions and 4 possible transitions, if errors have occurred at random, the Ts/Tv ratio for random false positive errors ($TSTV_\epsilon$) should be 0.5, that is, $\frac{TS_f}{TV_f} = 0.5$, assuming no systematic sequencing error biases.

Solving this system of four equations, it follows that $TV_f = \frac{TSTV_T \times TV_o - TS_o}{TSTV_T - 0.5}$ and $TS_f = 0.5 \times TV_f$, so the false discovery rate, $\frac{TS_f + TV_f}{TS_o + TV_o}$, can be estimated as:

$$\frac{TS_f + TV_f}{TS_o + TV_o} = \frac{0.5\left(\frac{TSTV_T \times TV_o - TS_o}{TSTV_T - 0.5}\right) + \frac{TSTV_T \times TV_o - TS_o}{TSTV_T - 0.5}}{TS_o + TV_o}$$

Assuming the true Ts/Tv ratio ($TSTV_T$) is between 2.0 and 2.1, by this calculation we estimate a false discovery rate of 0.1-2.9% among the BRIDGES ERVs.

Supplementary Note 3. Potential sources of bias among ERVs

3.1. Motif-specific error rates

Certain sequence motifs may be more susceptible to sequencing error, which could lead to a non-random distribution of false positive singleton calls and subsequently bias our analyses[4,5]. Allhoff et al. (2013)[5] reported context-specific errors for the Illumina HiSeq platform, noting that the most common of these are strand-specific T>N errors at 5'-GGGT-3' motifs (i.e., there is no evidence of an excess of

A>N errors at the reverse complement 5'-ACCC-3' motifs). We reason that if the BRIDGES ERVs are enriched for such context-specific errors, we should see significantly more T>N ERVs at the 5'-GGGT-3' motif than A>N ERVs at the 5'-ACCC-3' and motif. Of the 127,831 ERVs that occur at this motif, 63,861 were 5'-[A>N]CCC-3' variants, and 63,970 were 5'-GGG[T>N]-3' variants; this difference was not significant, indicating there is no evidence for an enrichment of T>N ERVs at this error-prone motif (exact binomial test; P=0.67). Allhoff et al. remark that the variants called at error-prone positions tended to have low base quality scores as well as significant strand bias, both of which are detectable with standard filtering protocols[5]. We therefore assume that most motif-specific errors are filtered by the default strand-bias and quality filters used in our variant calling pipeline, and any undetected errors have a negligible impact on our calculation of relative mutation rates and downstream analyses.

3.2. Mapping error

We expect the majority of ERVs in our data are mapped with high confidence, as the pre-filtering steps in our variant calling pipeline remove sites occurring on reads with average phred-scaled mapping quality score (MQ) <20 and/or where more than 10% of reads were ambiguously mapped (MQ0>10). This filtering strategy is similar to the filters employed by other large-scale sequencing projects that have demonstrated well-controlled error rates among singleton calls[6,7]. Because mapping errors are more likely to occur in highly-repetitive regions, such as centromeric and pericentromeric loci[8], including these regions in our analyses might bias our estimates of motif-specific mutation rates and/or the impact of genomic features. However, excluding these regions entirely might have detrimental side effects: dropping ERVs in these regions will reduce the precision of our estimates, and removing hard-to-map regions might preclude our ability to assess mutation patterns unique to these regions, as they may have many levels of heterogeneous overlap with genomic features.

To determine if excluding repeat-rich regions systematically influenced our inferred rates, we compared the 7-mer relative mutation rates estimated from the full, unfiltered set of ERVs with 7-mer rates estimated if we only count ERVs and reference motifs within the 1000 Genomes strict accessibility mask, which delineates the most uniquely mappable regions of the genome (covering ~72% of non-N

bases). These two sets of estimates were very well-correlated: within-type correlations were >0.96, indicating the estimated rates were highly consistent regardless of whether hard-to-map regions were removed (**Supplementary Fig. 6a**). Moreover, subtypes with larger differences between the two estimates tended to have fewer ERVs (**Supplementary Fig. 6b**), suggesting that most observed discrepancies might simply be an artifact of reduced precision among rare mutation classes.

When we applied the masked rates to predict the set of *de novo* mutations, we found these estimates had worse predictive performance than the unmasked estimates (**Table 1**). This result leads us to conclude that aggressively filtering for the highest-confidence call set comes at a cost of substantially reducing the precision of the relative mutation rate estimates, and potentially causing greater bias by ignoring the information captured by ERVs in the masked regions. Although we cannot entirely exclude the possibility of mapping error biases among the unmasked estimates, the benefits of having more numerous singletons across more contiguous genomic regions in the unmasked data outweigh the concerns about errors caused by poor mapping quality.

3.3. Mispolarization of ERVs

While most singletons in the BRIDGES sample are the true derived allele, population genetic theory suggests that <1/N=0.014% of singletons in a sample are the ancestral allele, and hence subject to the same evolutionary biases we wish to avoid. These mispolarized singletons may be hard to detect, as we expect ~0.25% of all singletons to carry the same allele in human and chimpanzee due to parallel mutations that have occurred since splitting from a common ancestor. Intuitively, these parallel mutations are especially likely to occur in hypermutable loci, so removing the 0.25% "ancestral" alleles created by parallel mutation may create a bigger bias than including the 0.015% truly ancestral alleles.

To understand the impact of removing all putatively ancestral alleles, we used an ancestral genome inferred by 6-way primate alignment[9] to annotate each allele with the putative ancestral state. We identified 363,705 singletons (~1% of all singletons) where the alternative allele was the same as the ancestral allele, and recalculated 7-mer relative mutation rates after removing these putatively mispolarized singletons. We found that this polarization filter did not strongly affect estimated rates:

across all types combined as well as within each type, the rates before and after removal of these sites were nearly perfectly correlated (Spearman's r>0.999). Further, we found that only 9 of the 24,576 7-mer rates differed significantly after applying this filter, and the re-estimated rates for these 9 subtypes differed from the original rates by no more than 10%. More importantly, 8 of these 9 subtypes were hypermutable CpG>TpG subtypes, consistent with our intuition that many putatively mispolarized sites are in fact parallel mutations in the human and chimpanzee lineages.

As a final analysis of the potential effects of mispolarization on our estimates, we applied these filtered rates to predict the GoNL/ITMI *de novo* mutations in the same logistic regression framework used to compare other estimation strategies. Goodness-of-fit statistics indicated that the filtered rates predicted *de novo* mutations better than 7-mer rates estimated without the polarization filter (ΔAIC=298). However, when comparing goodness-of-fit between type-specific models, these differences largely disappeared, with seven types showing negligible differences in AIC (ΔAIC < 7), and the unfiltered rates had lower AIC for three of these (non-CpG C>T, CpG>GpG, and CpG>ApG). Only two types had differences in AIC greater than 10: A>T types were predicted slightly better by the filtered rates (ΔAIC=16), but CpG>TpG types were predicted better by the unfiltered rates (ΔAIC=22), suggesting the accuracy of the filtered rates is particularly affected by parallel mutations at hypermutable CpG sites. Given this lack of consistent type-specific improvement when applying the polarization filter, we performed all subsequent analyses using the full set of 35.6 million ERVs.

Supplementary Note 4. Curation of MAC10+-derived mutation rate estimates

A potential concern with comparisons between our ERV-derived mutation rate estimates and Aggarwala and Voight's 1000G-based estimates[10] is that discrepancies might be partially attributable to technical differences between the two samples, not necessarily because the 1000G estimates are based on ancestrally older SNVs. For a more direct comparison, we curated a set of higher-frequency SNVs found in the BRIDGES data, removing the possibility that the dissimilar estimates are a result of differences in sequencing platform, variant calling, QC methods, and sampled individuals.

Aggarwala and Voight's mutation rate estimates are based on 7,051,667 intergenic variants observed in N=379 Europeans from the 1000 Genomes Phase I study[10]. Aggarwala and Voight do not state the exact site frequency spectrum for the European intergenic variants, but claim 26% of intergenic variants in the 1000G Phase I African sample are singletons or doubletons[10]. Thus, it is reasonable to assume that >80% of European intergenic SNVs in the 1000G data occur at a frequency greater than 1/(379*2)=0.0013 (i.e., the sample MAF of a singleton in the 1000G sample). To obtain SNVs in the BRIDGES sample in a frequency range comparable to this, we selected all SNVs with a minor allele count ≥10 (MAF≥0.0014). We identified 12,088,037 MAC10+ variants in our data, from which we estimated 7-mer relative mutation rates. We compared these estimates to 1) a set of ERV-derived 7-mer estimates calculated after randomly downsampling to an equivalent number (12,088,037 ERVs), and 2) the 1000G estimates. These comparisons show that the MAC10+ estimates are more closely correlated with the 1000G estimates (**Supplementary Fig. 3**) than with the downsampled ERV-derived estimates (**Supplementary Fig. 4**). We also used the MAC10+ estimates to predict the GoNL/ITMI *de novo* mutations, and found that this model tended to perform comparably to the 1000G model (**Supplementary Table 5**).

Acknowledgements

The BRIDGES study was supported by R01 MH094145 to Michael Boehnke and Richard M. Myers and U01 MH105653 to Michael Boehnke. The collection and storage of cases and controls from the Centre for Addiction and Mental Health (CAMH) in Toronto and from the Institute of Psychiatry, Psychology and Neuroscience (IoPPN), King's College London in London, U.K. was supported by funding from GlaxoSmithKline, from the Canadian Institutes of Health Research to John B. Vincent, MOP-172013 (CAMH), and funding from the National Institute for Health Research (NIHR) Biomedical Research Centre at South London and Maudsley NHS Foundation Trust and King's College London (IoPPN). The views expressed are those of the author(s) and not necessarily those of the UK NHS, the NIHR or the UK Department of Health. Case and control collection was supported by Heinz C. Prechter Bipolar Research Fund at the University of Michigan Depression Center to Melvin G. McInnis (Prechter). Data and biomaterials were collected for the Systematic Treatment Enhancement Program for Bipolar Disorder (STEP-BD), a multi-center, longitudinal project selected from responses to RFP #NIMH-98-DS-0001, "Treatment for Bipolar Disorder" which was led by Gary Sachs and coordinated by Massachusetts General Hospital in Boston, MA with support from 2N01 MH080001-001. The Genomic Psychiatric Cohort wishes to acknowledge all the research participants in this cohort; the study was supported by U01 MH105641, R01 MH085548, R01MH104964. The MCTFR study was supported through grants from the National Institutes of Health DA037904, DA024417, DA036216, DA05147, AA09367, DA024417, HG007022, and HL117626.

Supplementary References

1. Lawrence, M. S. *et al.* Mutational heterogeneity in cancer and the search for new cancer-associated genes. *Nature* **499,** 214–8 (2013).

2. Costello, M. *et al.* Discovery and characterization of artifactual mutations in deep coverage targeted capture sequencing data due to oxidative DNA damage during sample preparation. *Nucleic Acids Res.* **41,** 1–12 (2013).

3. DePristo, M. A. *et al.* A framework for variation discovery and genotyping using next-generation DNA sequencing data. *Nat. Genet.* **43,** 491–498 (2011).

4. Minoche, A., Dohm, J. & Himmelbauer, H. Evaluation of genomic high-throughput sequencing data generated on Illumina HiSeq and Genome Analyzer systems. *Genome Biol.* **12,** R112 (2011).

5. Allhoff, M. *et al.* Discovering motifs that induce sequencing errors. *BMC Bioinformatics* **14 Suppl 5,** S1 (2013).

6. Lek, M. *et al.* Analysis of protein-coding genetic variation in 60,706 humans. *Nature* **536,** 285–291 (2016).

7. Walter, K. *et al.* The UK10K project identifies rare variants in health and disease. *Nature* **526,** 82–90 (2015).

8. Horvath, J. E. *et al.* Molecular structure and evolution of an alpha satellite/non-alpha satellite junction at 16p11. *Hum. Mol. Genet.* **9,** 113–23 (2000).

9. McVean, G. A. *et al.* An integrated map of genetic variation from 1,092 human genomes. *Nature* **491,** 56–65 (2012).

10. Aggarwala, V. & Voight, B. F. An expanded sequence context model broadly explains variability in polymorphism levels across the human genome. *Nat. Genet.* **48,** 349–355 (2016).

11. Kundaje, A. *et al.* Integrative analysis of 111 reference human epigenomes. *Nature* **518,** 317–330 (2015).

12. Koren, A. *et al.* Differential relationship of DNA replication timing to different forms of human mutation and variation. *Am. J. Hum. Genet.* **91,** 1033–1040 (2012).

13. Kong, A. *et al.* Fine-scale recombination rate differences between sexes, populations and individuals. *Nature* **467,** 1099–1103 (2010).

14. Guelen, L. *et al.* Domain organization of human chromosomes revealed by mapping of nuclear lamina interactions. *Nature* **453,** 948–51 (2008).

15. Wu, H., Caffo, B., Jaffee, H. A., Irizarry, R. A. & Feinberg, A. P. Redefining CpG islands using hidden Markov models. *Biostatistics* **11,** 499–514 (2010).

Supplementary Figures

a.

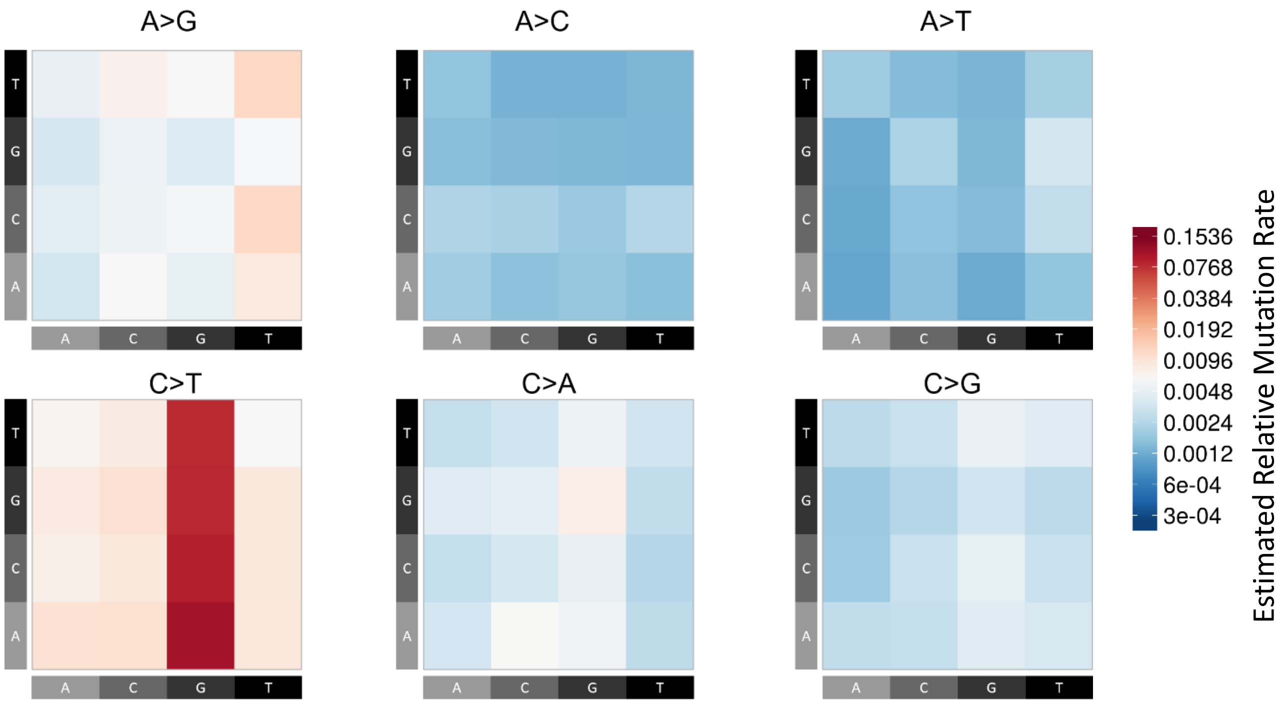

b.

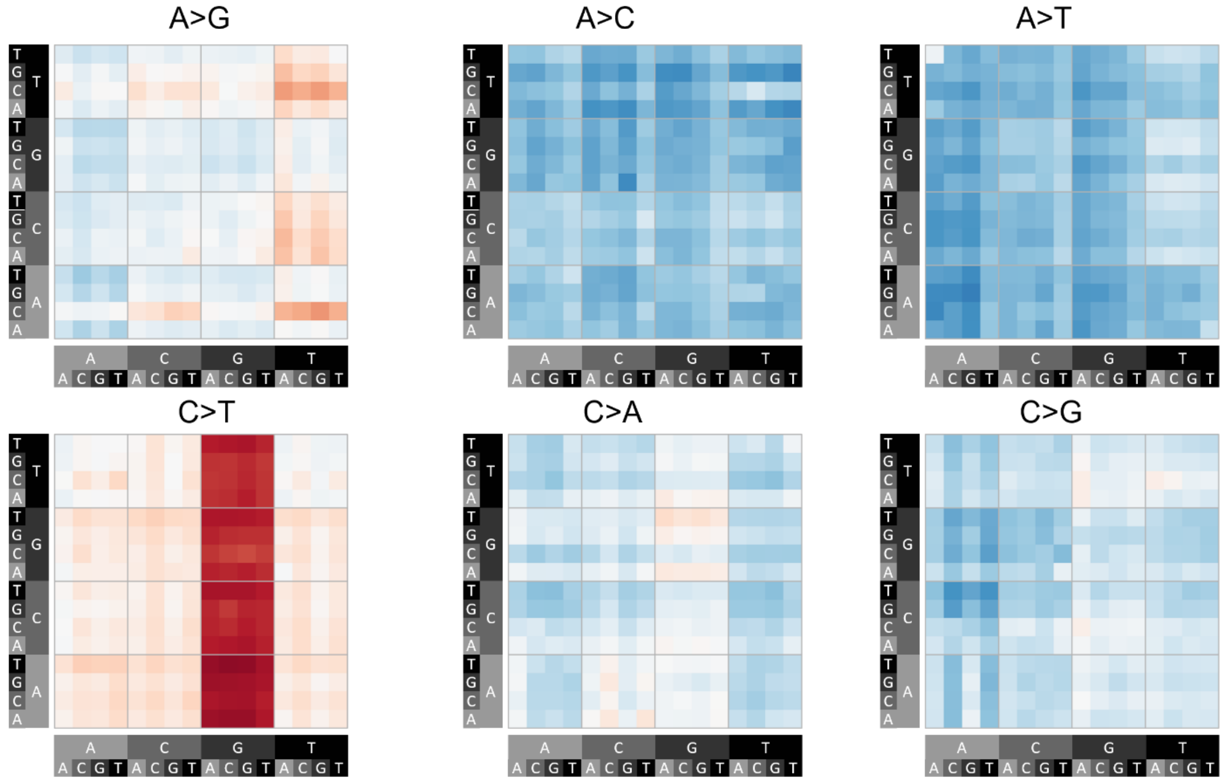

c.

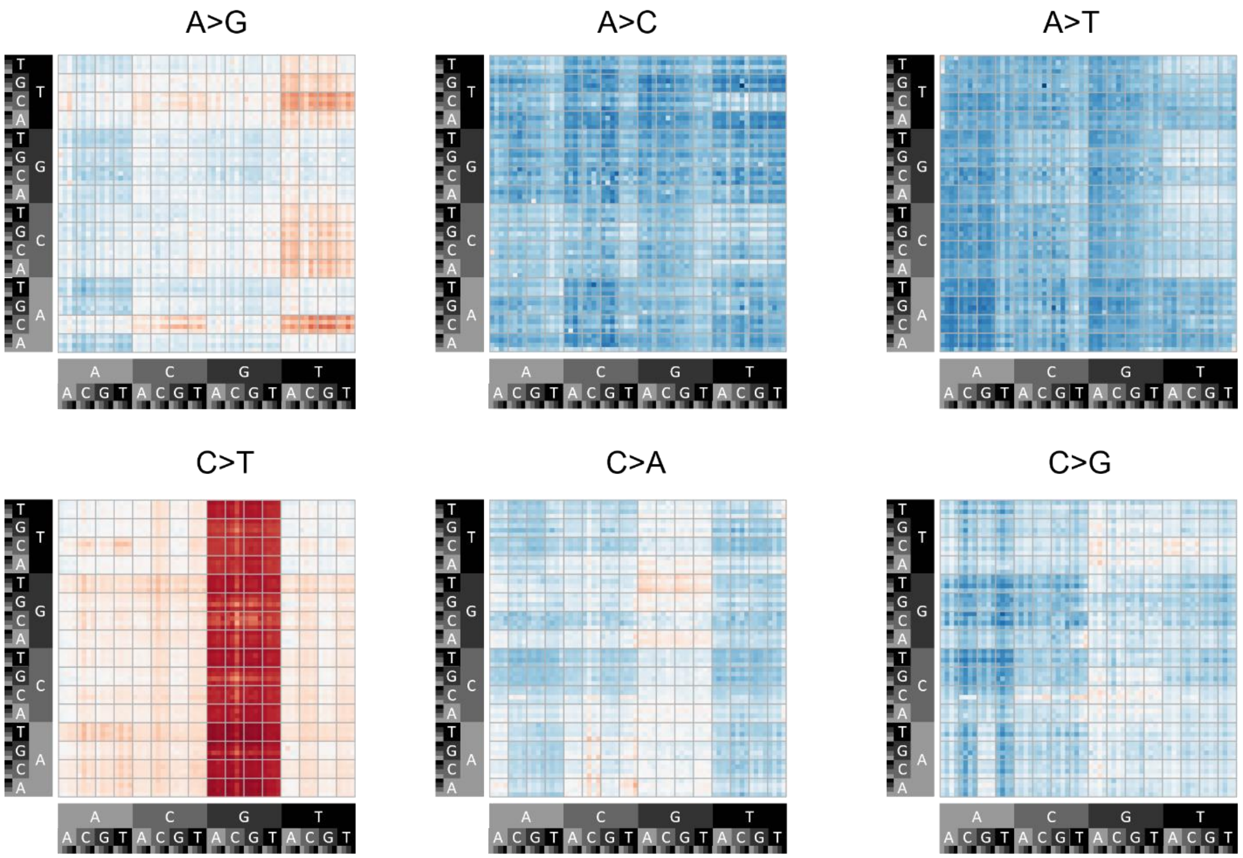

Supplementary Figure 1

High-resolution heatmaps of relative mutation rates for mutation subtypes up to a 7-mer resolution, estimated from the BRIDGES ERVs

(a) estimates for 3-mer mutation subtypes. **(b)** estimates for 5-mer mutation subtypes. **(c)** estimates for 7-mer mutation subtypes. Each cell delineates a subtype defined by the upstream sequence (y-axis) and downstream sequence (x-axis) from the central (mutated) nucleotide.

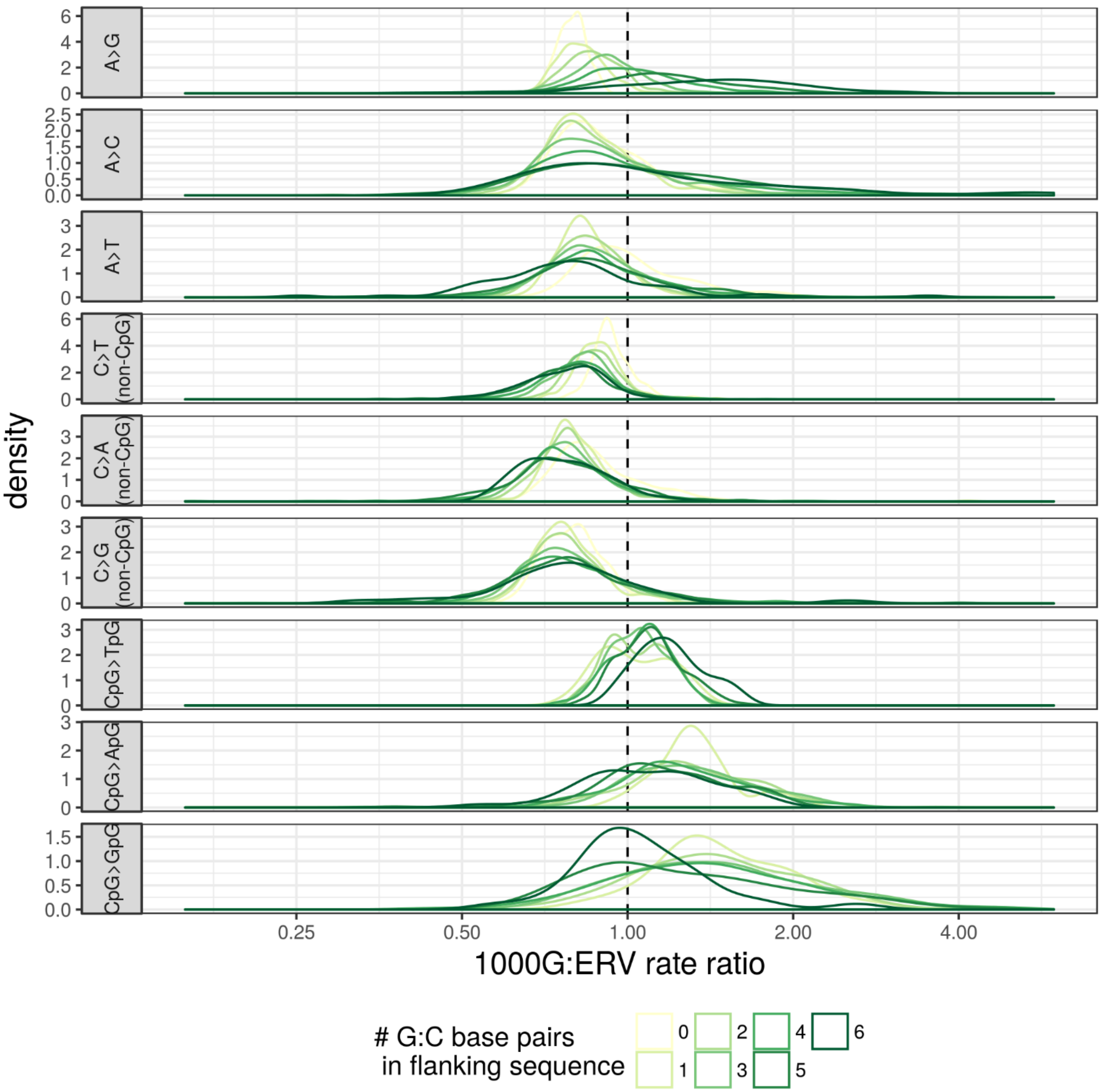

Supplementary Figure 2 Density plots comparing the distribution of ratios between the 1000G and ERV rate estimates

For each type, we grouped 7-mer subtypes by the number of G:C base pairs in the +/-3 flanking sequence, and plotted the distribution of ratios separately for each of these group. Mass to the right of the dashed line indicates estimated rates tend to be higher in the 1000G data, while mass to the left shows subtypes where estimated rates are higher in the BRIDGES ERV data.

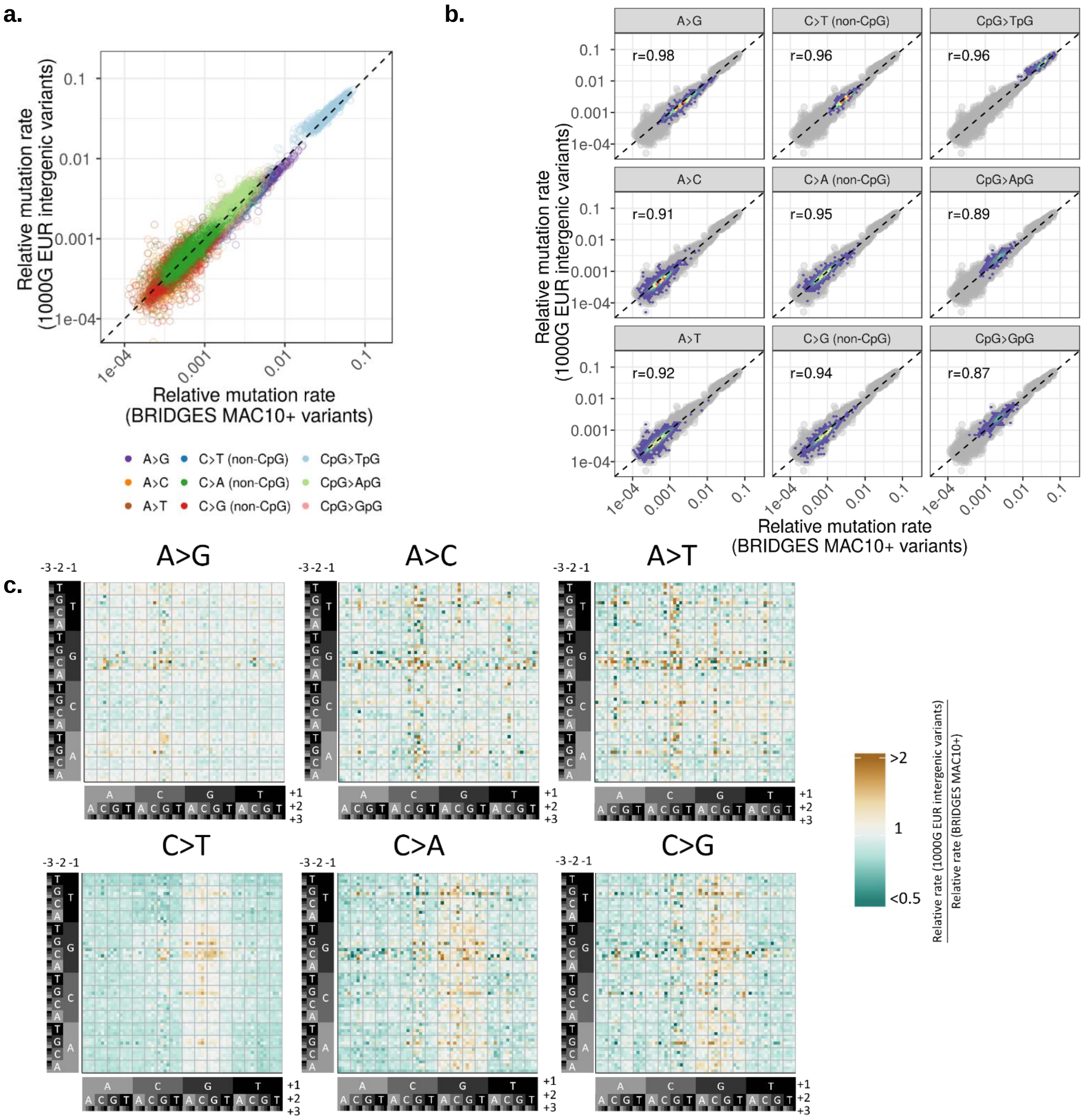

Supplementary Figure 3

Comparison of 7-mer relative mutation rates estimated from BRIDGES MAC10+ variants and 1000G Intergenic SNVs (a) Scatterplot of 7-mer subtype rates estimated from the BRIDGES MAC10+ data (x-axis), and 1000G intergenic SNV data (y-axis) **(b)** Type-specific 2D-density plots, as situated in the scatterplot of **a**. The dashed line indicates an expected least-squares regression line if there is no bias present. **(c)** Heatmap shows ratio between relative mutation rates calculated on MAC10+ variants and 1000G variants for each 7-mer mutation subtype. Subtypes with higher 1000G-derived rates relative to MAC10+-derived rates are shaded gold, and subtypes with lower 1000G-derived rates relative to MAC10+-derived rates are shaded green. 1000G-derived rates shown here are scaled relative to the MAC10+-derived rates.

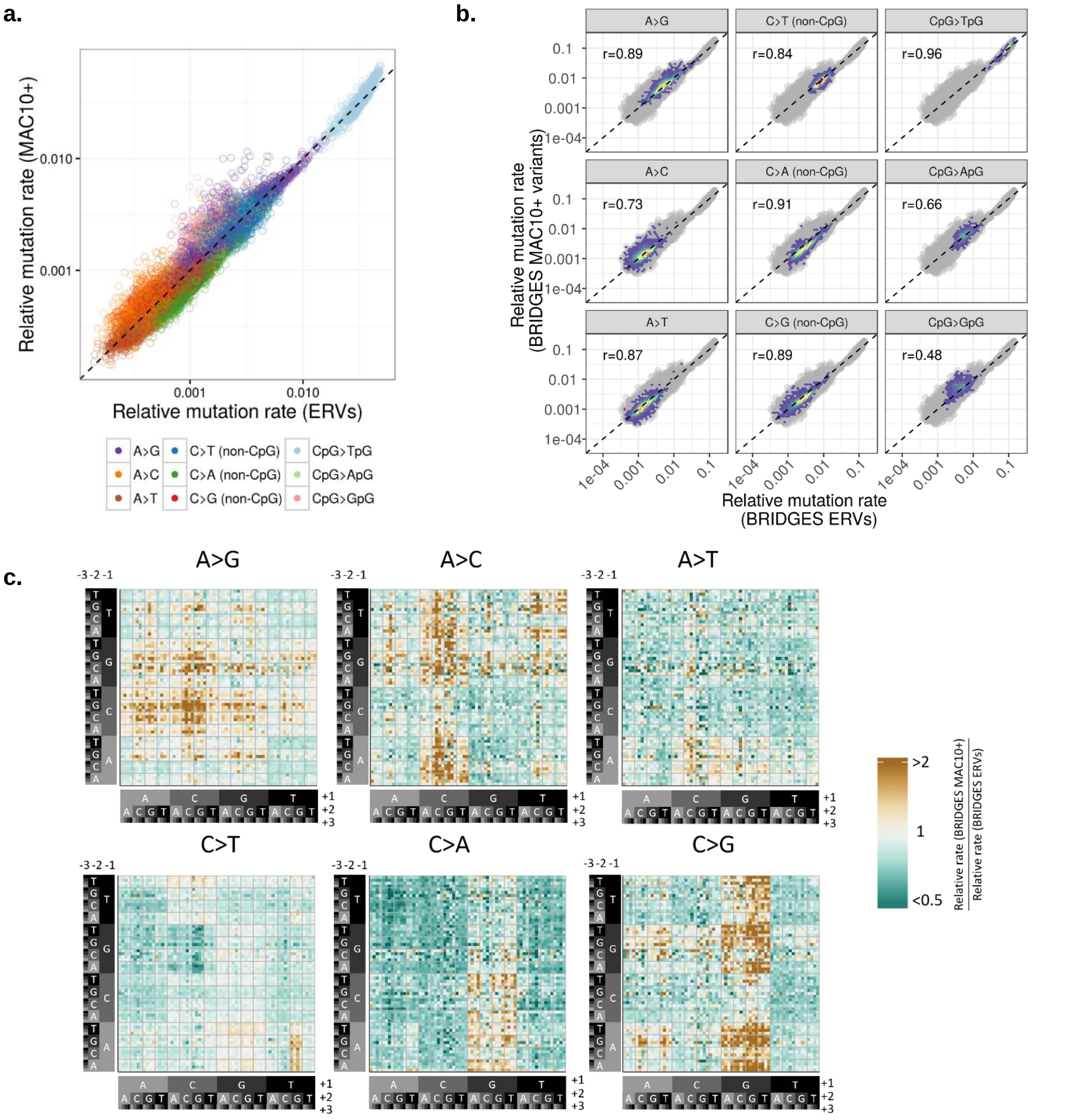

Supplementary Figure 4

Comparison of 7-mer relative mutation rates estimated from BRIDGES ERVs and BRIDGES MAC10+ variants (a) Scatterplot of 7-mer subtype rates estimated from the BRIDGES ERV data, after randomly downsampling the ERVs to 12,088,037 (x-axis) and the BRIDGES MAC10+ data (y-axis). **(b)** Type-specific 2D-density plots, as situated in the scatterplot of **a**. The dashed line indicates an expected least-squares regression line if there is no bias present. **(c)** Heatmap shows ratio between relative mutation rates calculated on MAC10+ variants and ERVs for each 7-mer mutation subtype. Subtypes with higher MAC10+-derived rates relative to ERV-derived rates are shaded gold, and subtypes with lower MAC10+-derived rates relative to ERV-derived rates are shaded green.

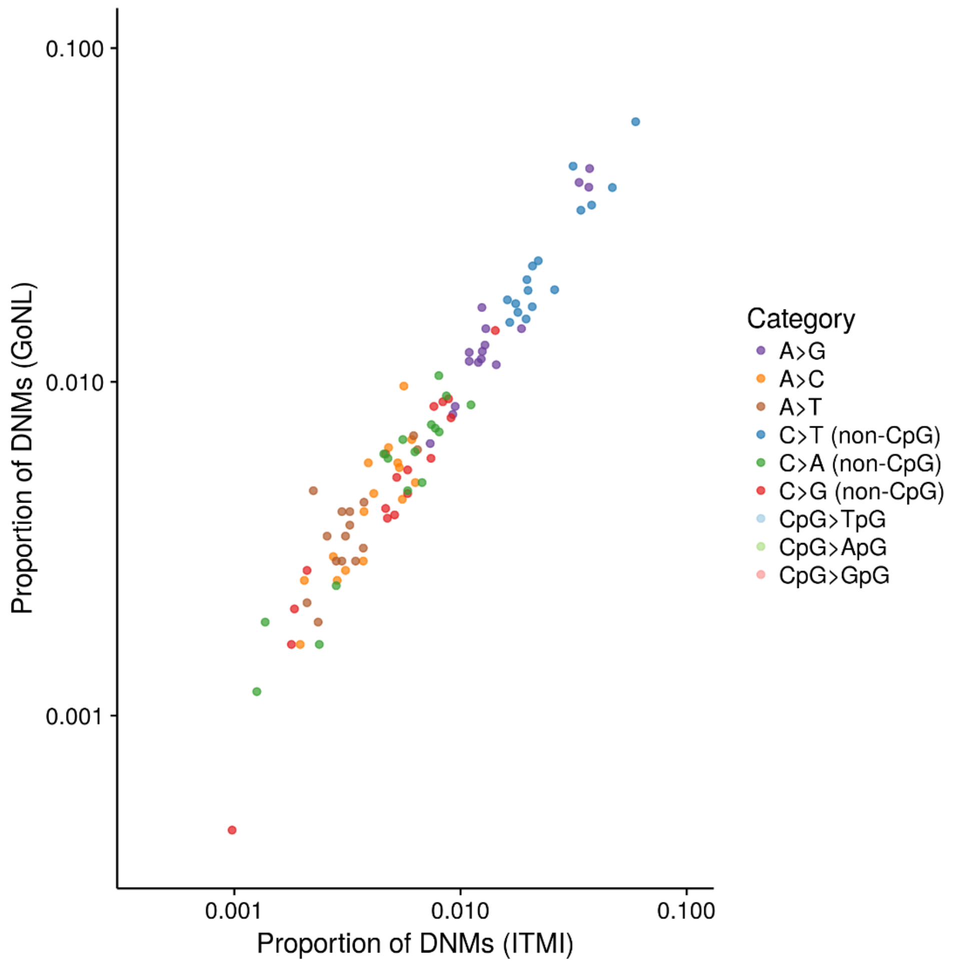

Supplementary Figure 5

Similar mutation spectra of the GoNL and ITMI data

Scatterplot shows the 3-mer mutational spectra (i.e., the proportion of all mutations falling within each of the 96 3-mer subtypes), calculated among *de novo* mutations from the ITMI (x-axis) GoNL (y-axis) trio sequencing studies.

a.

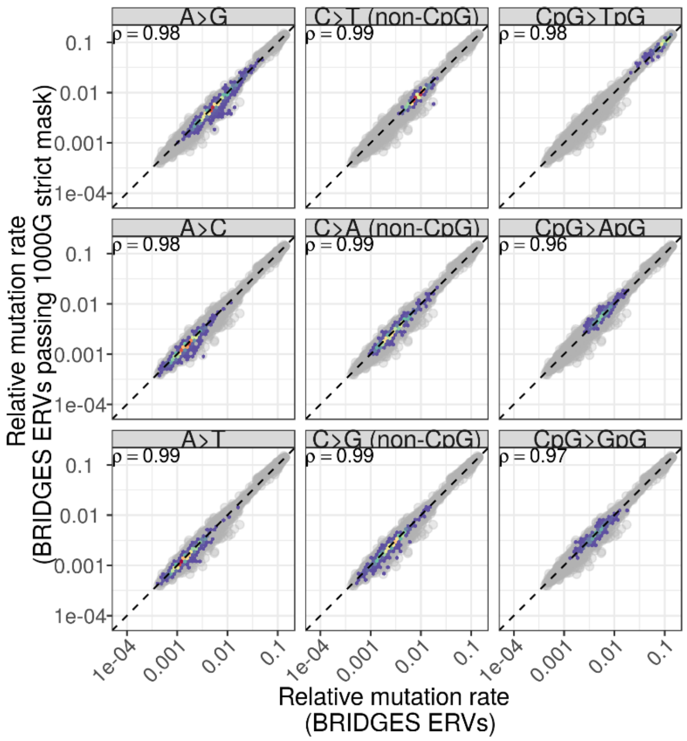

b.

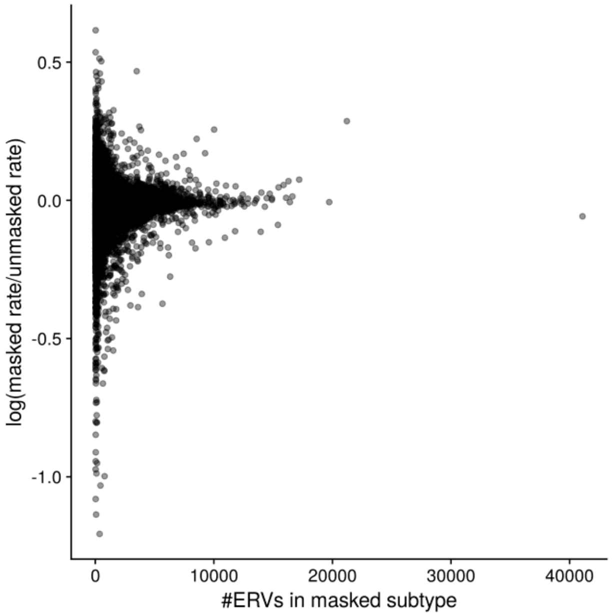

Supplementary Figure 6

Genome-wide estimates for ERV-based 7-mer subtypes are consistent with estimates from ERVs restricted to uniquely-mappable regions

(a) Relationship between masked and unmasked 7-mer relative mutation rate estimates, separated by type. **(b)** Relationship between number of ERVs per subtype (x axis) and discordance between the masked and unmasked rates, measured as the log ratio between the estimates (y axis).

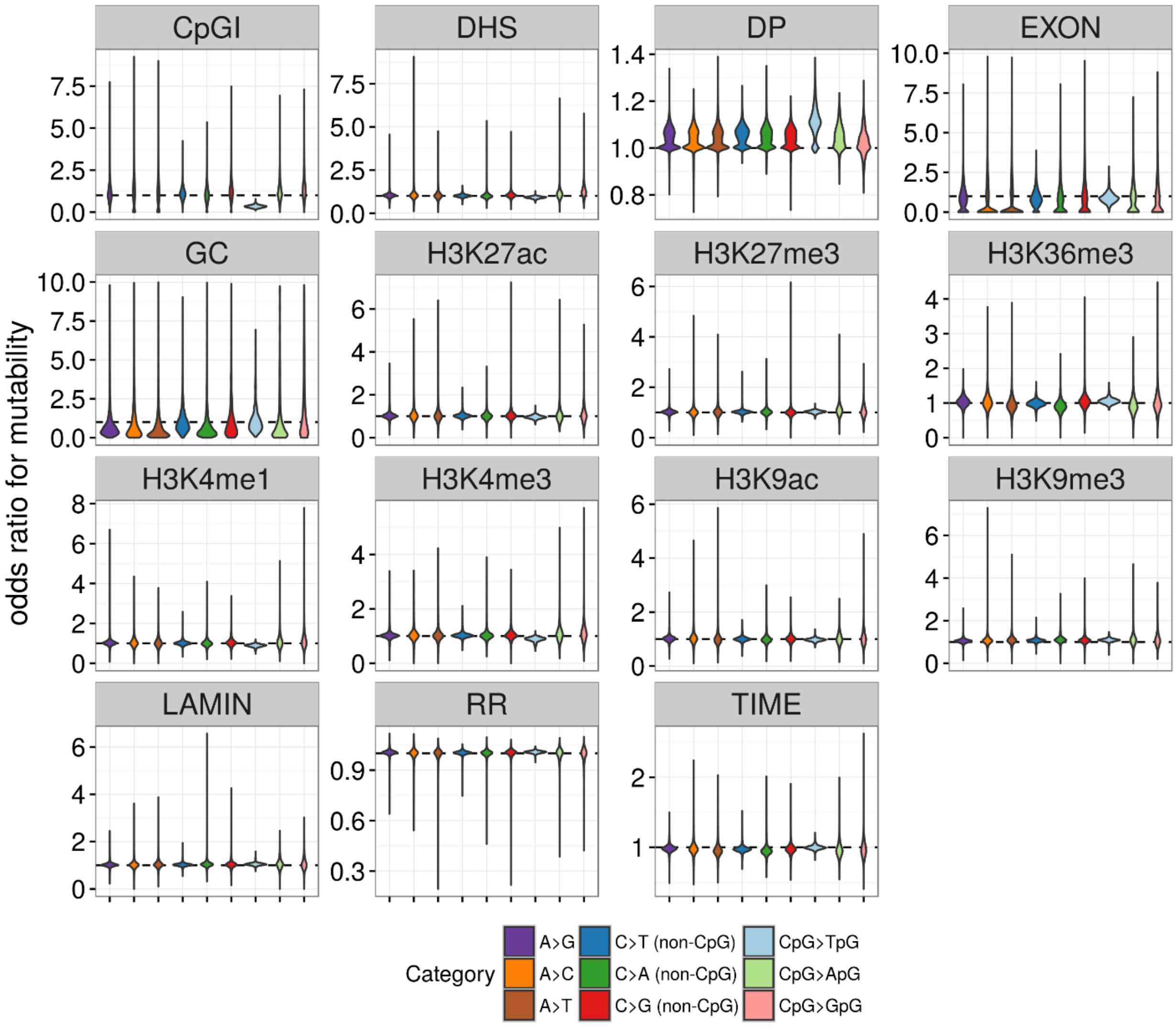

Supplementary Figure 7

Distributions of effect sizes on mutability for 14 genomic features and depth of sequencing

For each feature, we plotted the empirical distributions of the subtype-specific odds ratios for each basic mutation type, as estimated by our logistic regression models. *Replication timing is coded with negative values indicating later replicating regions, so an OR<1 means mutation rate increases in late-replicating regions.

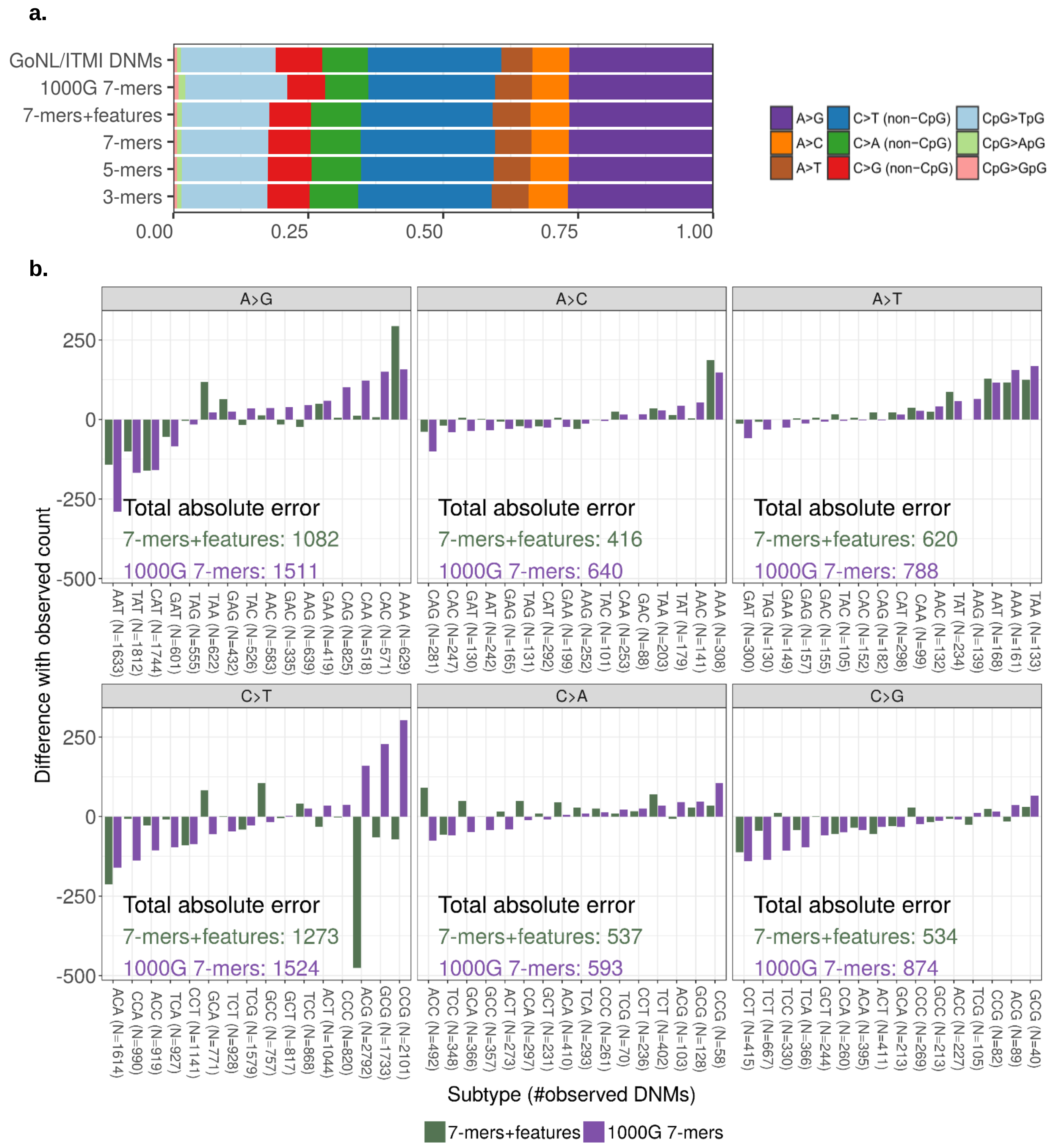

Supplementary Figure 8

Predicted mutation distributions under ERV-based models are more accurate than 1000G model
(a) Distribution of the GoNL/ITMI *de novo* mutations across basic mutation types compared to the distributions predicted under the 1000G 7-mer model and each of the BRIDGES ERV-based models. **(b)** Difference between model-predicted and observed number of mutations per 3-mer subtype for the 7-mer+features model (green bars) and 1000G 7-mer model (purple bars). The number of observed mutations for each subtype is indicated along the x-axis. In each panel, subtypes are sorted in increasing order of differences under the 1000G 7-mers model.

Supplementary Tables

Supplementary Table 1 **Quality comparison between filtered partitions of BRIDGES singletons**

Partition	# Singletons	Ts/Tv ratio	%dbSNP (b142)	% of Full Set
Full Set	35,574,417	2.00	17.4	100
Filter 2 (MQ>56)	33,550,098	2.01	17.3	94
Filter 3 (passed 1000G strict mask)	26,810,791	1.97	17.5	75
All Filters (MQ>56, 1000G strict mask)	16,535,856	2.00	17.6	46

Supplementary Table 2 **t-tests for differences in mean 1000G/ERV ratio of GC-poor vs. GC-rich 7-mer motifs**

Type	Mean 1000G/ERV ratio (≤3 C/G bases)	Mean 1000G/ERV ratio (≥4 C/G bases)	P-value
A>C	0.97	1.12	8.00e-30
A>G	1.00	1.28	2.37e-161
A>T	0.89	0.89	0.81
C>A (non-CpG)	0.76	0.72	2.61e-09
C>G (non-CpG)	0.89	0.93	2.98e-04
C>T (non-CpG)	0.93	0.85	1.75e-39
CpG>ApG	1.15	0.96	4.97e-22
CpG>GpG	1.46	1.33	2.80e-04
CpG>TpG	1.02	0.98	1.01e-09

For each mutation subtype, we calculated the ratio between 1000G-derived and ERV-derived relative mutation rates. Then, for each of the 9 basic types, we grouped 7-mer subtypes into low C/G subtypes (≤3 C/G bases in the +/-3 flanking positions) and high C/G subtypes (≥4 C/G bases in the +/-3 flanking positions) and performed t-tests for differences in the mean 1000G/ERV ratios of these two groups.

Supplementary Table 3 **Comparison of observed and simulated goodness-of-fit for *de novo* prediction models under different sized non-mutated backgrounds**

Model	Observed		Simulated		Background size
	AIC	R^2	AIC	R^2*	
1-mers	292542	.109	272925	.185	500,000
3-mers	284889	.139	241863	.299	
5-mers	282995	.146	239672	.307	
7-mers	282491	.148	238967	**.310**	
7-mers (BRIDGES MAC10+ SNVs)	283599	.144	240434	.304	
7-mers (1000G intergenic SNVs)	284764	.139	241724	.300	
1-mers	353896	.088	344108	.117	1,000,000
3-mers	335319	.118	317322	.197	
5-mers	332861	.124	315400	.202	
7-mers	332321	.126	314760	**.204**	
7-mers (BRIDGES MAC10+ SNVs)	342886	.103	316791	.198	
7-mers (1000G intergenic SNVs)	344003	.100	317953	.195	
1-mers	416998	.072	414016	.080	2,000,000
3-mers	404738	.102	392367	.132	
5-mers	402853	.107	390698	.136	
7-mers	402375	.108	390051	**.138**	
7-mers (BRIDGES MAC10+ SNVs)	404378	.103	392509	.132	
7-mers (1000G intergenic SNVs)	405523	.100	393741	.129	
1-mers	454267	.066	452950	.069	3,000,000
3-mers	441042	.095	434665	.109	
5-mers	439153	.099	433243	.112	
7-mers	438700	.100	432517	**.114**	
7-mers (BRIDGES MAC10+ SNVs)	441059	.095	435270	.108	
7-mers (1000G intergenic SNVs)	442181	.092	436443	.105	

*The simulated R^2 of the best possible model for each background size, indicated in bold, represents the optimal performance we can expect.

Supplementary Table 4 **Comparison of model AIC considering only *de novo* mutations from the GoNL or ITMI study**

Model	GoNL DNMs (11,020 mutations)	ITMI DNMs (35,793 mutations)
1-mers	114945	288707
3-mers	111952	280025
5-mers	111507	278542
7-mers	111381	278201
7-mers (BRIDGES MAC10+ SNVs)	111913	279580
7-mers (1000G intergenic SNVs)	112185	280401

Models fitted to a background of 1 million non-mutated sites, as described previously. Note that the difference in AIC between the two datasets is due to the difference in number of DNMs and is not comparable between the GoNL and ITMI studies. Goodness of fit statistics for both datasets have the same rank order.

Supplementary Table 5 Type-specific model fit statistics for mutation rate estimation strategies applied to the *de novo* testing data. Each type is shown in a sub-table, with the number of *de novo* mutations and non-mutated sites used in the partitioned testing data indicated in the subheading.

A>C (2920 *de novo* mutations; 198481 non-mutated sites)

Model	Nagelkerke's R^2	AIC
3-mers	0.002	32831
5-mers	0.007	32701
7-mers	0.009	32641
7-mers+features	0.009	32636
7-mers (downsampled BRIDGES ERVs)	0.008	32670
7-mers (BRIDGES MAC10+ SNVs)	0.003	32809
7-mers (1000G intergenic SNVs)	0.004	32775

A>G (11400 *de novo* mutations; 198793 non-mutated sites)

Model	Nagelkerke's R^2	AIC
3-mers	0.039	91474
5-mers	0.065	89455
7-mers	0.068	89212
7-mers+features	0.069	89111
7-mers (downsampled BRIDGES ERVs)	0.064	89505
7-mers (BRIDGES MAC10+ SNVs)	0.061	89732
7-mers (1000G intergenic SNVs)	0.061	89746

A>T (2455 *de novo* mutations; 198320 non-mutated sites)

Model	Nagelkerke's R^2	AIC
3-mers	0.015	28130
5-mers	0.016	28114
7-mers	0.016	28106
7-mers+features	0.016	28105
7-mers (downsampled BRIDGES ERVs)	0.007	28350
7-mers (BRIDGES MAC10+ SNVs)	0.001	28498
7-mers (1000G intergenic SNVs)	0.003	28463

non-CpG C>A (3620 *de novo* mutations; 128765 non-mutated sites)

Model	Nagelkerke's R^2	AIC
3-mers	0.012	35362
5-mers	0.022	35039
7-mers	0.03	34794
7-mers+features	0.032	34743
7-mers (downsampled BRIDGES ERVs)	0.029	34823
7-mers (BRIDGES MAC10+ SNVs)	0.024	35000
7-mers (1000G intergenic SNVs)	0.027	34892

non-CpG C>G (3561 *de novo* mutations; 128746 non-mutated sites)

Model	Nagelkerke's R^2	AIC
3-mers	0.006	35889
5-mers	0.018	35490
7-mers	0.024	35321
7-mers+features	0.024	35321
7-mers (downsampled BRIDGES ERVs)	0.023	35350
7-mers (BRIDGES MAC10+ SNVs)	0.019	35480
7-mers (1000G intergenic SNVs)	0.018	35489

non-CpG C>T (10321 *de novo* mutations; 128774 non-mutated sites)

Model	Nagelkerke's R^2	AIC
3-mers	0.005	79879
5-mers	0.012	79502
7-mers	0.014	79379
7-mers+features	0.014	79353
7-mers (downsampled BRIDGES ERVs)	0.013	79395
7-mers (BRIDGES MAC10+ SNVs)	0.012	79487
7-mers (1000G intergenic SNVs)	0.013	79434

CpG>ApG (304 *de novo* mutations; 6108 non-mutated sites)

Model	Nagelkerke's R^2	AIC
3-mers	0.014	2788
5-mers	0.024	2767
7-mers	0.027	2763
7-mers+features	0.029	2761
7-mers (downsampled BRIDGES ERVs)	0.025	2763
7-mers (BRIDGES MAC10+ SNVs)	0.022	2771
7-mers (1000G intergenic SNVs)	0.025	2762

CpG>GpG (270 *de novo* mutations; 6292 non-mutated sites)

Model	Nagelkerke's R^2	AIC
3-mers	0.013	2560
5-mers	0.015	2557
7-mers	0.022	2545
7-mers+features	0.026	2538
7-mers (downsampled BRIDGES ERVs)	0.015	2556
7-mers (BRIDGES MAC10+ SNVs)	0.015	2556
7-mers (1000G intergenic SNVs)	0.011	2564

CpG>TpG (6960 *de novo* mutations; 6289 non-mutated sites)

Model	Nagelkerke's R^2	AIC
3-mers	0.011	20321
5-mers	0.02	20232
7-mers	0.025	20173
7-mers+features	0.06	19777
7-mers (downsampled BRIDGES ERVs)	0.024	20182
7-mers (BRIDGES MAC10+ SNVs)	0.027	20151
7-mers (1000G intergenic SNVs)	0.027	20148

Supplementary Table 6 Genomic features used in mutation models

Feature	Source	Cell Type	Resolution
H3K4me1, H3K4me3, H3K9ac, H3K9me3, H3K27ac, H3K27me3, H3K36me3	Roadmap Epigenomics Project[11]	Peripheral Blood Mononuclear Primary Cells	1bp (inside vs. outside of broad peak)
Replication timing	Koren et al., 2012[12]	Lymphoblastoid	1kb window
Recombination rate	Kong et al., 2010[13] (deCODE sex-averaged recombination rate map)	--	10kb window
Lamin B1 domains	Guelen et al., 2008[14]	Tig3ET normal human embryonic lung fibroblasts	1bp (inside vs. outside of LAD)
DNase hypersensitivity sites	ENCODE	multiple	1bp (inside vs. outside of DHS region)
Exonic site	RefSeq gene database	--	1bp (inside vs. outside of exon)
CpG island	Wu et al., 2010[15]	--	1bp (inside vs. outside of CpG island)
% GC content	Calculated from reference genome	--	10kb

A script to download the exact external data files used in this paper is available at https://github.com/carjed/smaug-genetics

Supplementary Table 7 Chi-squared tests for enrichment or depletion of *de novo* mutations occurring in feature-associated subtypes

Feature	Expected direction of effect	*de novo* relative mutation rate		p-value
		[a]Inside feature	[b]Outside feature	
H3K9me3†	Increased	1.98E-05	1.73E-05	**4.87E-05**
High Recombination rate (> 2)	Increased	3.66E-05	3.43E-05	0.18
H3K27me3†	Decreased	5.44E-06	3.14E-06	0.99
H3K27ac	Decreased	1.22E-04	1.23E-04	0.50
Exons	Decreased	1.20E-04	8.66E-05	0.99
H3K4me1	Decreased	1.10E-04	1.40E-04	**1.84E-10**
H3K4me3†	Decreased	1.00E-04	1.50E-04	**4.92E-23**
H3K9ac†	Decreased	1.49E-05	7.49E-06	0.99
Lamin-associated domains	Increased	6.91E-05	7.46E-05	0.75
High GC content (> 0.55)	Decreased	1.23E-05	9.74E-06	0.82
	Increased	1.14E-05	4.65E-06	**6.61E-04**
H3K36me3	Decreased	4.73E-06	6.14E-06	**2.59E-03**
	Increased	1.99E-05	1.51E-05	**5.50E-10**
CpG Islands	Decreased	3.68E-05	1.60E-04	**5.00E-117**
	Increased	5.39E-06	6.69E-06	0.79
Late replication timing (< -1.25)*	Increased	6.18E-06	5.48E-06	**0.026**
Early replication timing (> 1.25)*	Increased	1.55E-05	8.06E-06	**2.25E-02**
DHS	Decreased	5.03E-05	3.08E-05	0.99
	Increased	1.75E-05	1.21E-05	**4.92E-04**

Significant differences that are consistent with the expected direction of effect are indicated by a one-sided p-value in bold. †Four features had associations in the opposite direction, but these predicted effects could not be tested due to a lack of *de novo* mutations observed within the associated subtypes. *Some subtypes showed a significant *negative* association with replication timing, such that the mutation rate would be higher in *early-* rather than late-replicating regions, so we tested these subtypes separately.
